# Human Pluripotent Stem Cell-Derived Alveolar Organoids for Gene-editing and Lung Adenocarcinomas Modeling

**DOI:** 10.7150/ijbs.118304

**Published:** 2025-10-01

**Authors:** Lian Li, Yuchen Liu, Lifan Liu, Shanshan Zhao, Jianqi Feng, Jiayi Zhou, Yanqun Zhang, Xiaohang Shen, Xinlong Wang, Kechen Chen, Jie Lv, Kaican Cai, Shuan Rao, Zhili Rong, Ying Lin

**Affiliations:** 1 Cancer Research Institute, School of Basic Medical Sciences, State Key Laboratory of Multi-organ Injury Prevention and Treatment, Guangdong Province Key Laboratory of Immune Regulation and Immunotherapy, Southern Medical University, Guangzhou 510515, China; 2 Huizhou Central People's Hospital Postdoctoral Innovation Practice Base, Southern Medical University, Huizhou 516008, China; 3 Department of Thoracic Surgery, Nanfang Hospital, Southern Medical University, Guangzhou 510515, China

**Keywords:** alveolar organoids, long-term expandability, genetic engineering, lung adenocarcinoma

## Abstract

Human pluripotent stem cell (hPSC)-derived alveolar organoids have emerged as valuable tools for studying lung development, modeling pulmonary diseases, and drug discovery, though their application has been hindered by laborious differentiation protocols and technical complexity. Here, we present an hPSC-derived alveolar organoid (hALO) system with exceptional long-term expandability (>30 passages), efficient cryopreservation resilience, and streamlined production achieved through earlier 3D culture initiation and elimination of cell sorting requirements. Transcriptomic analysis across passages confirmed hALOs contain alveolar progenitors and AT2 lineages, recapitulating pseudoglandular-to-canalicular development while partially maintaining adult AT2 immune-related functions. The system permits alveolar epithelial differentiation via pharmacological modulation of WNT/YAP signaling or through orthotopic transplantation, while multiplex genetic engineering enables programmable disease modeling and adenocarcinoma pathogenesis studies. These versatile capabilities establish hALOs as a robust dual-phase platform for mechanistic investigation of lung epithelial biology and disease modeling across* in vitro* and *in vivo* environments.

## Introduction

Alveoli are the basic respiratory units of the mammalian lung. Diseases associated with alveolar dysfunction impose significant healthcare burdens worldwide. For instance, lung cancer persists as the foremost contributor to cancer-related mortality worldwide^1^, idiopathic pulmonary fibrosis (IPF) is a progressive and incurable disease affecting millions globally^2^, ^3^, chronic obstructive pulmonary disease (COPD) ranks as the third leading cause of death worldwide^4^, while infectious diseases such as SARS-CoV-2-associated pneumonia have triggered global pandemics^5^. These persistent clinical challenges highlight the critical necessity for developing physiologically relevant in vitro models that can accurately recapitulate pulmonary architecture, facilitate disease pathogenesis studies, and enable rigorous assessment of emerging therapeutic interventions.

As self-organized 3D structures aggregated by stem cells and/or their progeny *in vitro*, organoids recapitulate structural and functional features of their *in vivo* counterparts, serving as powerful complementary models to traditional 2D cell lines, genetically engineered mouse models (GEMMs), and patient-derived xenografts (PDXs)^6^-^8^. Alveolar organoids (ALOs) can be established from either pluripotent stem cell (PSC) sources, encompassing embryonic stem cells (ESCs) and induced pluripotent stem cells (iPSCs)^9^-^15^, or adult stem cells (ASCs) including primary alveolar type 2 (AT2) cells and human embryonic lung epithelial tip populations^16^-^18^. ASC-derived ALOs exhibit high reproducibility and simplified culture protocols due to their lineage commitment^19^, ^20^. However, limitations including restricted access to human tissues, limited long-term expansion of primary cells, and donor-to-donor variability hinder the utility of ASC-derived organoids^21^. To overcome these constraints, more scalable PSC-derived ALOs have been developed to address the scarcity of primary human alveolar samples^7^, ^21^.

The generation of hPSC-derived ALOs depends on stepwise differentiation of hPSC into definitive endoderm (DE), anterior foregut endoderm (AFE), “ventralized” AFE (VAFE), lung progenitors (LPs), and ultimately hALOs^11^-^14^. This protracted differentiation process (25-80 days) limits large-scale applications^10^-^12^, ^22^. Furthermore, the successful generation of hALOs largely depends on the quality and variability of hPSCs^23^, ^24^. Maintaining high-quality hPSCs demands substantial technical expertise and resources, while differentiation efficiency varies significantly across cell lines and batches^25^. Additional limitations include cellular immaturity compared to adult lungs and contamination by off-target cell types^26^, ^27^, which collectively impede widespread adoption of hPSC-based organoid models.

To overcome these limitations, developing next-generation organoid systems with enhanced long-term expandability, cryostability, and post-thaw viability has become imperative. Complementary strategic refinement of culture conditions—particularly for alveolar type 1 (AT1) lineage specification and transplantation compatibility—could achieve higher-order biophysical relevance and functional specification^14^, ^28^-^31^. While current differentiation paradigms enable generation of hPSC-derived lung organoids^10^-^12^, their longitudinal culture robustness, cryobanking feasibility, resuscitation efficiency, and most critically, genetic engineering flexibility have not been systematically investigated in prior studies. Our research endeavors to rectify this by conducting a detailed investigation into these crucial aspects. Furthermore, the majority of current protocols rely on purifying cells based on surface markers like CPM^high^, CD47^high^/26^low^, or using lineage reporters such as NKX2-1^eGFP^ and SFTPC^tdTomato^, yielding relatively pure progenitors or AT2^11^, ^12^, ^32^. While effective, these strategies may compromise physiological complexity and introduce technical hurdles.

In this study, we efficiently generated and systematically characterized human ALOs (hALOs) derived from multiple PSC lines through non-enriched differentiation protocols. Our hALOs exhibited robust expandability (>30 passages) while maintaining alveolar identity, achieved efficient cryopreservation with full functional recovery across all passages, and underwent differentiation through pharmacological modulation of WNT/YAP signaling or orthotopic transplantation. Transcriptomic analysis confirmed hALOs contain alveolar progenitors and AT2 cells, recapitulating fetal lung development from pseudoglandular to canalicular stages while maintaining authentic AT2 signatures and key immune elements often lost during cell sorting. Versatile genetic engineering was achieved through targeted *NKX2-1* activation, *ACTB* locus transgene integration, and *TP53* knockout, establishing programmable disease modeling capabilities. Notably, orthotopic transplantation of KRAS^G12D^-mutated hALOs reconstituted early lung adenocarcinoma histopathological features, functionally validating their utility for mechanistic studies of pulmonary disease pathogenesis.

## Materials and Methods

### Maintenance of hPSCs

The H9 hESC line was obtained from WiCell Research Institute. The iPSC line (YiPS-1, induced from somatic cells of testes) and H1 hESC line were kind gifts from Dr.Xiaoyang Zhao (Department of Development, Southern Medical University). Cells were maintained in mTeSR1 medium (STEMCELL Technologies; cat. no. #85851 and #85852) on 6-well tissue culture plates coated with Matrigel (BD Biosciences; cat. no. 354277) with the medium changed daily. Cells were passaged by digestion with TrypLE Express (Gibco, Grand Island, NY, USA) at 1:10-1:15 split ratios every 4 days. The use of both ES and iPSC lines was approved by the Biomedical Ethics Committee of Southern Medical University.

### Generation and maintenance of human alveolar organoids

hPSCs were stepwise differentiated into alveolar organoids using our previously described protocol with brief modifications^14^, ^22^. Briefly, to induce DE, hESCs or hiPSCs were seeded on 24-well tissue culture dishes and cultured for 3 days in RPMI1640 medium containing 100 ng/ml activin A (R&D Systems; cat. no. 338-AC-050) and 2 µM CHIR99021 (Tocris Bioscience; cat. no. 4423-10MG). To generate AFE in day 4-7, the medium was replaced with Advanced DMEM/F12 (Life Technologies; cat. no. 12634010) supplemented with 200 ng/ml Noggin (R&D Systems; cat. no. 6057-NG-100), 500 ng/ml fibroblast growth factor 4 (FGF4) (Peprotech; cat. no. 100-31-1MG), 10 µM SB431542 (Tocris Bioscience; cat. no. 1614-10MG), and 2 µM CHIR99021. Cells were embedded in Matrigel (BD Biosciences; cat. no. 356237) for 3D culture on day 8. Then, VAFE was generated by treating cells with 20 ng/ml human bone morphogenetic protein 4 (BMP4) (R&D Systems; cat. no. PRD314-10), 0.5 µM all-trans retinoic acid (Sigma-Aldrich, St. Louis, MO, USA; cat. no. R2625), 3.5 µM CHIR99021 in Dulbecco's Modified Eagle's Medium (DMEM)/F12 (Life Technologies; cat. no. 11320033) from day 8-14. To generate LP, VAFE-enriched cells were cultured in DMEM/F12 supplemented with 10 ng/ml human FGF10 (R&D Systems; cat. no. 345-FG-025), 10 ng/ml human keratinocyte growth factor (KGF) (Novoprotein; cat. no. CM88), 3 µM CHIR99021, and 20 µM DAPT (Sigma-Aldrich; cat. no. D5942) from day 15-21. For hALOs induction and maintenance, the cells were incubated in Ham's F12 (Gibco; cat. no. 21127022) with 50 nM dexamethasone (Sigma-Aldrich; cat. no. D4902), 100 µM 8-Br-cAMP (MCE; cat. no. HY-12306), 100 µM 3-isobutyl-1-methylxanthine (Wako; cat. no. 095-03413), 10 ng/ml KGF, 3 µM CHIR99021, 10 µM SB431542, 1% B-27 supplement, 0.25% bovine serum albumin (BSA) (Sigma-Aldrich; cat. no. A1470), 15 mM HEPES (Sigma-Aldrich; cat. no. H0887), 0.8 mM CaCl2 (Sigma-Aldrich; cat. no. C3881), and 0.1% ITS premix (Corning; cat. no. 354351). Organoids were passaged either in a cluster or in single-cell as described previously^14^. Particularly, when there were increasingly dead cells among large ALOs, organoids could be passaged by single-cell to promote the outgrowth of proliferating cells.

### Cryopreservation and resuscitation of organoids

Organoids can be successfully cryopreserved either as clusters or as single cells. For cryopreservation, cells were resuspended at a concentration of 1 × 10^6^ cells (or 4-6 drops of organoids) in 1 ml of freeze medium (90%FBS+10%DMSO) and frozen in a cryovial. For organoid thawing, cells were added to a conical tube containing DMEM/F12 to dilute the DMSO. Then organoids were centrifuged to remove the supernatant, washed by DPBS, and embedded in Matrigel to start 3D culture. After gel solidification, alveolar medium with Rho Kinase inhibitor Y-27632 (MCE, cat. no. HY-10583) was added to culture the organoids. After 48h, cells could be cultured with medium without Y-27632.

### Organoid transfection

Organoids were first digested into single cells by TrypLE Express (Gibco; cat. no. 12604-021). A total of 10,000 to 20,000 cells were used for every transduction. For electroporation, cells were resuspended in 100 µl of BTXpress High Performance Electroporation Solution (BTX, cat. no. 45-0802) and 15 μg of plasmids (5 μg pCAG-SB100×, 10 μg pT3-CAG-mNeonGreen) were supplemented, the mixture was then added into a 2mm cuvette and electroporation was performed on ECM 830 SQUARE WAVE ELECTROPORATION SYSTEM (BTX) using the following conditions: Poring Pulse (Voltage=175 V, Pulse Length=5 ms, Pulse Interval=50 ms, Number of Pulse=2), Transfer Pulse (Voltage=20 V, Pulse Length=50 ms, Pulse Interval=50 ms, Number of Pulse=5). After electroporation, cells were fed with complete medium with Y-27632, recovered for 30 min at room temperature (RT) and finally seeded in Matrigel. For lentivirus-based transduction, cells were resuspended in complete medium with Y-27632 and high titer Lenti-CAG-mNeonGreen or Lenti-EF1α-mCherry (MOI varied from 2 to 5) supplemented with 4 μg/mL polybrene were added into the medium. The organoid-virus mixture was transferred into 24-well culture dish and incubated for 2 h at 37 °C in a culture incubator to allow transduction. Cells were then washed with DPBS and seeded in Matrigel. For Polyethylenimine (PEI) transfection, cells were resuspended in complete medium with Y-27632 and transferred into 24-well culture dish. 4 μg of plasmid (1.33 μg pCAG-SB100×, 2.66 μg pT3-CAG-mNeonGreen) and 16 μL of 1 μg/μL PEI were mixed in Opti-MEM and then added into the dish for 4 h to allow transduction. Cells were washed with DPBS and seeded in Matrigel after transduction.

### Flow cytometry

Organoids were digested into single cells by TrypLE Express, stained with DAPI, washed and resuspended in DPBS, and strained into FACs tubes. Flow Cytometry was performed using a BD LSRFortessa™ and data were analyzed by FlowJo.

### Gene activation, knock-in, knock-out and overexpression in hALOs

For NKX2-1 activation, H9 hESC expressing Cas9-P300 after the induction of doxycycline^33^ was used and differentiated into hALOs, and a short gRNA targeting the promoter region of NKX2-1 was designed and cloned into pU6- gRNA 2.1 scaffold (gRNA sequence: 5′-GCCCCCGCAGCTCA-3′). pU6- eGFP gRNA 2.1 scaffold (gRNA sequence: 5′- AGCACTGCACGCCGT -3′) was used as control. The PEI transfection protocol described above was used and 3 days after transfection the RNA was extracted for qRT-PCR.

To generate knock-in cell lines, 3 plasmids were constructed: one expressed gRNA targeting ACTB locus (pU6-ACTB-sgRNA, gRNA sequence:5′-AATATGAGATGCGTTGTTAC-3′), the second expressed Cas9 and the DNA-binding domain of Sleeping Beauty transposase (pCAG-Cas9-N57), the third was donor plasmid expressing fluorescent protein eGFP or mNeonGreen (PbluSK-gACTB-IR-puro-pCAG-d2mNeonGreen or PbluSK-gACTB-IR-puro-EF1α-EGFP). The PEI transfection protocol described above was used (0.66 μg pU6-ACTB-sgRNA, 1.33 μg pCAG-Cas9-N57 and 2 μg donor plasmid). One week after transfection, 1 μg/mL puromycin was used for selection. Selection was maintained until the WT organoids had completely died. Single surviving organoids that had been successfully transfected could be picked under fluorescence microscope to obtain a knock-in cell line.

To achieve TP53 knock-out, the Cas9-P300 expressing hALOs mentioned above were used. The plasmid Lenti-U6-TP53 sgRNA-EF1α-neo-WPRE (gRNA sequence:5′-GCACCCGCGTCCGCGCCA-3′) was constructed. Lentivirus was generated by transfection of HEK-293T cells with Lenti-U6-TP53 sgRNA-EF1α-neo-WPRE and the packaging plasmids psPAX2 and pMD2.G using PEI. hALOs were transfected using lentivirus-based transduction protocol mentioned above. One week after transfection, doxycycline was used to induce the expression of Cas9 to knock-out TP53, and 400 μg/mL G418 was added to the media to select the infected organoids. The mutation of DNA was detected by Sanger sequencing and knock-out efficiency was calculated using TIDE (http://shinyapps.datacurators.nl/tide/). For doxorubicin treatment, 1 μM doxorubicin (MedChemExpress; cat. no. HY-15142) was added into the medium of WT and P53 KO hALOs for 48 h and organoids were harvested for immunofluorescence.

For the overexpression of KRAS^G12D^, plasmid Lenti-EF1α-KRASG12D-HA-IRES-puro was a kind gift from Dr. Mengfeng Li's group. Plasmid pLKO.1-puro was served as control. Lentivirus transfection and puromycin selection were proceeded as described above. For morphological statistics, hALOs transfected with Lenti-EF1α-KRASG12D-HA-IRES-puro or pLKO.1-puro were digested into single cell and embedded in Matrigel in the density of 1500 cells/μL. Cells were allowed to grow for 10 days and the thickness of each hALO was measure by ImageJ software in microscope 10x field of view.

The primers used for genotyping were listed in Table S1.

### Tag-seq

We utilized our Tag-seq technique to detect CRISPR off-targets in P53 knock-out experiments^34^. Briefly, 350 ng of pCAG-Cas9-mcherry, 150 ng of Lenti-U6-TP53 sgRNA-EF1α-neo-WPRE plasmids, and 10 nM oligonucleotide Tag (Tag sequence: Tag-F: P-A*T*CTCTGAGCCTTATGCGAAATGCGTGTTATCG*C*A; Tag-R: P-T*G*CGATAACACGCATTTCGCATAAGGCTCAGAG*A*T. Note: P, phosphorylation; *, phosphorothioate linkage) were transfected by PEI in a 24-well plate. Cells were harvested 72 hours later and genomic DNA was extracted for library construction, sequencing, and performing bioinformatics analyses.

### RNA extraction and qRT-PCR

Total RNA was extracted using TRIzol reagent (Yeasen biotech; cat. no. 10606ES60), and was reverse transcribed using the Evo M-MLV RT Kit (Accurate Biology; cat. no. AG11711) according to manufacturer's guidelines. qRT-PCRs were performed using the SYBR Green Premix Pro Taq HS qPCR Kit (Accurate Biology; cat. no. AG11701). The relative expression level of each gene was normalized to that of glyceraldehyde 3-phosphate dehydrogenase (GAPDH). Three biological replicates of each sample were prepared, and data are presented as the mean ± SD. Primers used are listed in Table S2.

### RNA isolation and RNA-seq of organoids

Comparative RNA-seq and alignment were carried out as published previously^22^. Briefly, total RNA was isolated from organoids that were differentiated into P4, P13 and P20 using TRIzol reagent. Samples were sent to GENEWIZ company (www.genewiz.com.cn) for sequencing library preparation and RNA sequencing. Quality control of reads was conducted using FastQC (version 0.11.9, https://github.com/s-andrews/FastQC). Ligation adaptors were removed using TrimGalore (version 0.6.7, https://github.com/FelixKrueger/TrimGalore). The cleaned reads were aligned to the human reference genome (GENCODE, GRCh38/hg38) using STAR (version 2.7.9a)^35^. Only reads uniquely mapped to the human genome were used for downstream analysis, and gene-level expression was quantified based on read counts.

Differential gene expression analysis was performed using the DESeq2 package (version 1.34.0)^36^, with adjusted P values (p.adj) calculated using the Benjamini-Hochberg method. Gene variability across samples was assessed using the coefficient of variation (CV), calculated via the limma package (version 3.50.3)^37^. Principal component analysis (PCA) was performed using FactoMineR (version 2.6)^38^, and heatmaps were generated with pheatmap (version 1.0.12, https://github.com/raivokolde/pheatmap). Hierarchical clustering was conducted using the hclust function from the stats package (version 4.1.2). Gene Ontology (GO) and KEGG pathway enrichment analyses were carried out using clusterProfiler (version 4.2.2)^39^. RNA-seq data for hALOs are available in the GEO database (GSE240203).

### scRNA-seq data analysis

Cell heterogeneity, clustering, integration, and visualization: Preliminary data preprocessing was performed using SeekSoulTools (v1.2.2, http://seeksoul.seekgene.com/)^40^ with human reference genome (GENCODE, GRCh38/hg38). Data processing was primarily conducted with Seurat R package (v5.1.0)^41^. Poor-quality cells were excluded, specifically those with fewer than 200 or more than 7,500 expressed genes, based on the distribution of the data. Only genes expressed in three or more cells were used for further analysis. Cells were also discarded if their mitochondrial gene percentages were over 10%. Potentially cell doublets were removed by DoubletFinder (v2.0.4)^42^. Integration analysis was performed using the FindIntegrationAnchors and IntegrateData functions (dims = 1:20). The expression data were normalized and scaled with NormalizeData function (normalization.method = "LogNormalize", scale.factor = 10,000), and ScaleData function (model.use="linear"). The top 2,000 genes with the highest standardized variance were identified using FindVariableFeatures function (selection.method="vst"). Principal component analysis (PCA) was computed using RunPCA function with default parameters. Shared nearest neighbor (SNN) graph was computed using the FindNeighbors function, with the first 21 PCA dimensions as input. These dimensions were selected because their cumulative explained variance exceeds 90%. Cell clusters were defined using Louvain algorithm with the FindCluster function. For visualization uniform manifold approximation and projection (UMAP) was used. Pseudotime analysis was performed using monocle3 (v1.3.7, preprocess_cds num_dim = 25)^43^ to reconstruct the lineages of different cell types. A semi-supervised approach was used to determine the development of distinct cell types within hALOs, with *EPCAM^+^* cell types indicated. The differential expression of marker genes among the differentiated AT2 cells in hALOs was compared using pseudobulk analysis, with the wilcoxon signed-rank test employed for statistical evaluation. Differentiated AT2 time course sequencing data were analyzed using Tcseq (v1.28.0, algo='cm', k=10)^44^. The analysis identified five clusters with consistent trends in hALOs, and Gene Ontology analysis was conducted to explore the biological processes within these gene clusters. irGSEA (v3.3.2, method = ssgsea)^45^ and UCell (v2.8.0)^46^ were used to assess gene signature scoring for pathways related to AT2 maturation between differentiated AT2 and differentiated AT2 (hALOP20).

Comparison and correlation between our single-cell dataset and datasets of developmental stages: Our single-cell dataset and public single-cell dataset (E-MTAB-11278, GSE168191)^47^, ^48^ were integrated in Seurat using FindIntegrationAnchors and IntegrateData (dims=1:20). The combined datasets were embedded and visualized using UMAP, with cells labeled according to their original annotations. *EPCAM*^+^ cells were used to evaluate the correlation between hALOs and published fetal and adult lung data, with Pearson's correlation coefficients calculated based on the pseudobulk normalized expression of all genes.

Comparison of our single-cell dataset and datasets from other hPSC-derived platforms: For cross-platform comparisons, we processed our dataset and three published datasets (GSE150708, GSE162936, GSE148113)^49^-^51^ through standardized quality control and normalization. All datasets were then uniformly re-annotated using our established pipeline with consistent marker genes and Seurat clustering parameters to ensure direct comparability. Cell type proportions were derived from these harmonized annotations.

Single-cell RNA-seq data for our hALOs are available in the GEO database (GSE308817).

### Comparison and integration with public bulk RNA-seq datasets

Our dataset and four public datasets (GSE96642, E-MTAB-6023, GSE245721, GSE241335)^11^, ^30^, ^52^, ^53^ were integrated for comparative analysis. To ensure consistency and minimize technical variation, gene expression matrices were combined, and the top 2,000 most variable genes were selected based on their standard deviation across all samples. The resulting expression matrix was scaled and used for downstream clustering and correlation analysis. Differential expression and pathway enrichment analyses for the public datasets were performed using the same methods as described above.

### EdU incorporation

10 μM EdU (Life Technologies) was added into the medium of organoids and cultured for 2 h. The organoids were then washed and fixed, and EdU staining was carried out according to the manufacturer's protocol (Beyotime, cat. no. C0075S). Images were acquired by confocal microscope.

### AT1 induction in hALOs

For the induction of AT1 by CHIR withdrawal (CHIR-), hALOs at early passage (P1-P2) were first cultured in alveolar medium mentioned above and then changed in medium without CHIR99021 for 7 days. Then the thickness of organoid was measured by imageJ and organoids were harvested for qRT-PCR or immunofluorescence. To generate AT1 by nuclear YAP activation, hALOs at any passage were used. Medium was replaced with Lats-IN-1 medium, in which 10 μM Lats-IN-1 (MedChemExpress, cat. no. HY-138489) was added while CHIR99021, SB431542 and KGF were removed in alveolar medium. hALOs were allowed to grow for 9 days and then harvested for qRT-PCR or whole-mount immunofluorescence.

### Air-liquid interface (ALI) culture

For ALI culture, 6.5 mm Transwell inserts (Corning; Cat. No. 3470) were coated with Matrigel (BD Biosciences) and incubated at 4°C overnight. hALOs were dissociated into single cells as previously described. Cells were resuspended in 200 μL of either ALOs medium, CHIR- medium, or Lats-IN-1 medium, then seeded onto the Transwell inserts at densities of 70,000 (high density) or 35,000 (low density) cells per insert. The cultures were maintained under submerged conditions for 2-3 days, during which 10 μM Y-27632 was added to the medium. Following this period, the apical medium was removed to establish ALI conditions, and cultures were continued for an additional 9 days.

### Transepithelial electrical resistance (TEER) measurement

TEER was measured using a Millicell® ERS 3.0 Digital Voltohmmeter (Millipore Sigma; Cat. No. MERS03000). Electrodes were sterilized by immersion in 70% isopropyl alcohol followed by DPBS rinsing. Prior to measurements, 200 μL and 500 μL of medium were added to the apical and basal chambers, respectively. For each sample, stabilized readings were recorded twice. The final TEER values were calculated by subtracting the Matrigel-coated blank well resistance from the mean sample resistance and multiplying by the growth area.

### Transmission electron microscopy

TEM was performed as previously described^22^. In brief, hALOs were collected and fixed in 2.5% glutaraldehyde, washed with 0.1 mol/L Phosphate buffer and further fixed with 1% osmium tetroxide. Then, the samples were washed with phosphate buffer and dehydrated with 30%, 50%, 70%, 80%, 85%, 90%, 95% and 100% alcohol sequentially. After infiltration with different mixtures of acetone/Epon (2:1, 1:1, v/v), the samples were embedded in pure Epon and polymerized at 60 °C for 48 h. Ultrathin sections (80-100 nm) were cut on Ultramicrotome (Leica EMUC7), put on grids and stained with uranyl acetate and lead citrate. After washing and drying, images were acquired by the digital camera on TEM (FEI, Tecnai G2 20 TWIN, 200 kv).

### Bleomycin injury and *in vivo* orthotopic transplantation

Bleomycin injury and *in vivo* orthotopic transplantation were performed as described previously^14^. Briefly, one week before transplantation, B-NDG mice (NOD. CB17-*Prkdc^scid^Il2rg^tm1^*/Bcgen, Biocytogen) were anesthetized and lungs were damaged by intratracheal instillation with 1 U/kg bleomycin in a 20-μL volume (with 10-20% body weight loss at 7 days post-treatment serving as a quality control metric). On the day of transplantation, organoids were digested into single cells (>90% viability by trypan blue exclusion) as described above; the cells were diluted to a concentration of 100,000/20 μL in sterile DPBS and administered to anesthetized mice via intratracheal injection. Lungs were harvested 5 months after transplantation. All experiments involving mice were approved by the Institutional Animal Care and Use Committee of Southern Medical University (IACUC approval number: L2019018).

### Immunofluorescence

For paraffin sectioning, as described previously^14^, organoids or lung tissue was fixed in 4% paraformaldehyde and dehydrated in 30% sucrose solution. Then the samples were embedded and cut at a thickness of 6-10 μm. Samples were permeabilized for 30 min in 0.2% Triton X-100 (Sigma-Aldrich) and blocked in 5% BSA at RT for 1 h. The sections were then incubated overnight at 4°C with primary antibodies, washed 3 times with PBS, incubated with secondary antibodies at RT for 1 h, washed 3 times with PBS, and counterstained with 4′,6-diamidino-2-phenylindole (Sigma-Aldrich; cat. no. D9542) for 5 min, before imaging with the Nikon A1 confocal microscope. Antibodies used are listed in Table S3.

For whole-mount immunofluorescence, organoids were isolated from Matrigel using Cell Recovery Solution (Corning; 354253) at 4 °C for 30 min, washed with PBS, and then moved into the glass bottom microwell dishes (Corning, P35G-0-20-C), fixed with 4% paraformaldehyde overnight at 4 °C, washed 3 times with PBS, permeabilized and blocked using 0.2% Triton X-100 and 5% BSA at RT for 1 h. Primary antibodies were incubated overnight at 4 °C, washed 3 times with PBS, and then stained with secondary antibodies at RT for 45 min. Nuclear counterstained with DAPI for 5 min. Organoids were mounted and imaged using the confocal microscope. The NIS-Elements software was used to process the images and for the 3D reconstruction. Antibodies used are listed in Table S3.

### Hematoxylin & Eosin (H&E) staining and Immunohistochemistry

As described previously, lung tissues were fixed with 4% paraformaldehyde, dehydrated by standard protocol, and then embedded with paraffin. 5 μm sections were obtained, H&E and immunohistochemistry staining was carried out in accordance with the standard protocol. Antibodies used are listed in Table S3. This study utilizing human lung adenocarcinoma (LUAD) and lung squamous cell carcinoma (LSCC) samples was approved by the Medical Ethics Committee of Nanfang Hospital, Southern Medical University (Approval No. NFEC-202108-K7). All samples were collected with prior written informed consent from the patients.

### Statistical analysis

Unless otherwise specified in figure legends, unpaired Student's t-tests were used for comparisons between two groups, while one-way ANOVA with Tukey's multiple comparisons test was employed for comparisons among three or more groups. All analyses were performed using Prism 8 software (GraphPad, La Jolla, CA, USA), with statistical significance set at p<0.05 unless noted otherwise.

## Results

### Scalable production and cryostability of hALOs

**Figure 1:**
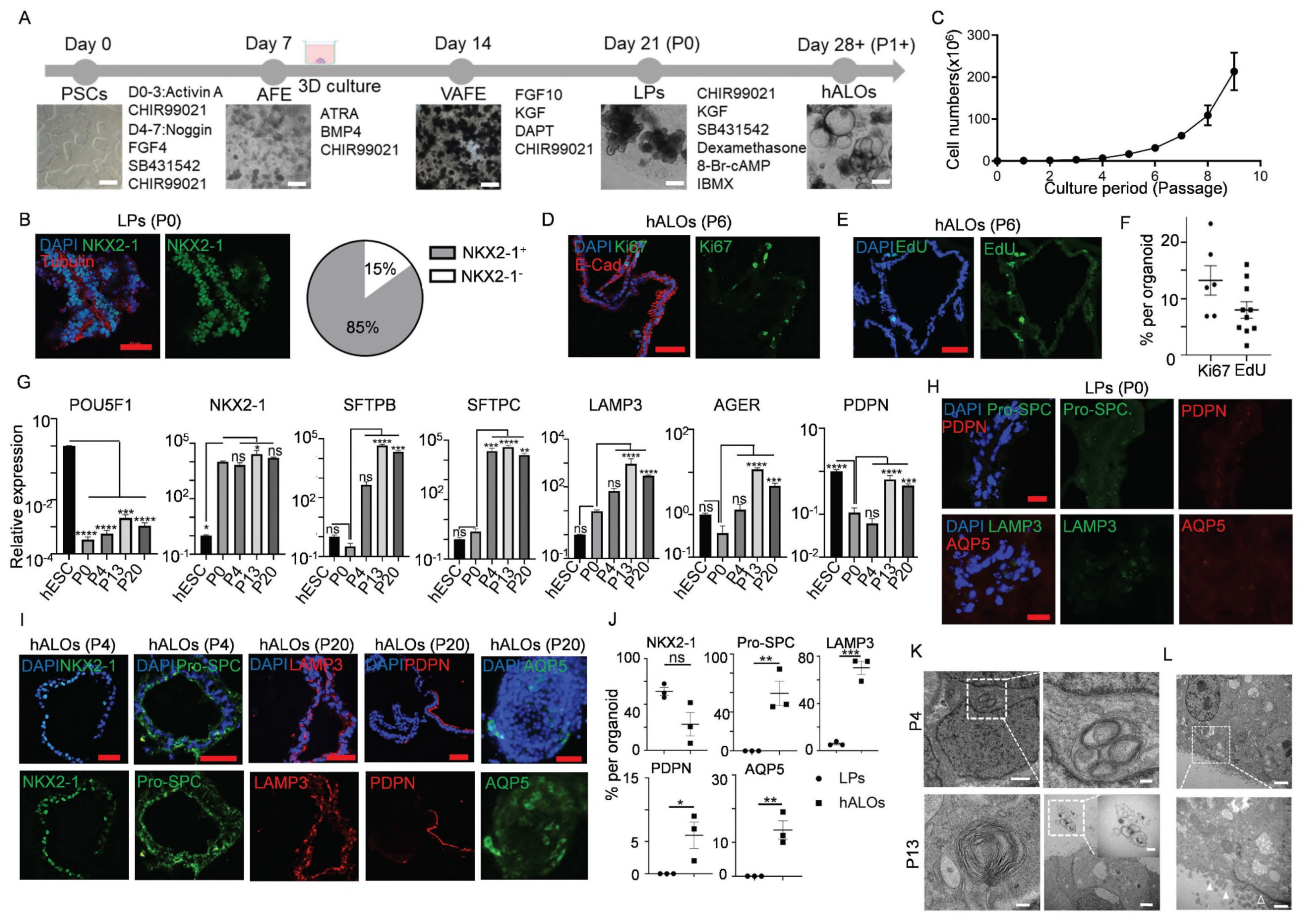
** Generation and long-term expansion of human alveolar organoids (hALOs).** (A) Schematic diagram of the stepwise differentiation protocol from hPSCs to hALOs, with corresponding phase-contrast micrographs illustrating morphological progression at key developmental milestones. Scale bars, 500 μm. (B) Immunofluorescence staining and quantification of NKX2-1 in organoids at day 21 of differentiation. Scale bars, 50 μm. (C) Growth curves of cultured hALOs were analyzed at the first 10 passages. (D, E) The proliferation of hALOs was determined by Ki67 (D) and EdU (E) staining. Scale bars, 50 μm. (F) Quantification of Ki67 or EdU positive cells in (D) and (E). (G) mRNA expression levels of differentiation markers. * p<0.05, ** p<0.01, *** p <0.001, **** p<0.0001 (one-way ANOVA compared to hESC or P0 hALOs as indicated in the figure, n=3). (H) Representative immunofluorescence labeling of LPs staining for indicated markers. Scale bars, 50 μm. (I) Representative immunofluorescence labeling of hALOs at different passages staining for indicated markers. Scale bars, 50 μm. (J) Quantification of immunofluorescence-positive cells in LPs and hALOs from three biological repicates. * p<0.05, ** p<0.01, *** p <0.001 (unpaired, two-tailed Student's t-test). (K) Transmission electron microcopy (TEM) analysis of P4 and P13 hALOs. Lamellar bodies (LBs) were indicated. Scale bars, 1 μm (P4 left panel), 200 nm (P4 right panel), 100 nm (P13 left panel), 2 μm (P13 right panel), 500 nm (P13 right panel inserted). (L) TEM analysis showing microvilli (solid arrowhead) and tight junctions (hollow arrowhead). Scale bars, 2 μm (top), 500 nm (bottom).

Building upon our prior differentiation platform with optimized parameters, we successfully generated hALOs from H9 hESCs through a staged induction protocol (Figure 1A)^14^, ^15^, ^22^, ^31^. Quantitative analysis of the lung lineage marker NKX2-1 at differentiation day 21 (P0) revealed expression in approximately 85% of cells, validating the efficiency and consistency of our protocol (Figure 1B). Remarkably, hALOs demonstrated sustained expandability with >800-fold numerical increase by passage 9 (0.25 ± 0.01 ×10⁶[P0] vs. 213.31 ± 25.80 ×10⁶ cells[P9]) (Figure 1C). Ki67 staining and EdU labeling showed that about 10% of the cells were in proliferation (Figure 1D-F). qRT-PCR showed that pluripotent marker *POU5F1* was downregulated, lung lineage marker *NKX2-1*, AT2 markers *SFTPB, SFTPC* and *LAMP3*, AT1 markers *AGER* and *PDPN* were upregulated after long-term culture (Figure 1G). Consistent with mRNA expression patterns, immunofluorescence analysis of both sectioned and whole-mount samples demonstrated robust alveolar-related protein expression, with P0 lung progenitors (LPs) serving as an appropriate negative control by showing negligible expression of both AT2 and AT1 markers (Figure 1H-J). Even after 241 days-expansion (30 passages), organoids remained normal morphologies and expressed alveolar-related markers (Figure S1A-F). Transmission electron microcopy (TEM) confirmed that hALOs at different passage (P4 and P13) formed lamellar bodies (LBs), the functional organelles of AT2. Particularly, we found that LBs were secreted into the lumen of organoids at P13, which is a hallmark of AT2 maturation^54^ (Figure 1K). Besides, typical characteristics of epithelial differentiation, such as microvilli on the apical of the organoids and tight junctions on the cell membrane, were found by TEM (Figure 1L). Additionally, hALOs could be generated from multiple PSC lines including H1 hESCs and iPSCs, demonstrating broad applicability of our system, though quantitative immunofluorescence revealed lineage-dependent efficiency: iPSC-derived hALOs showed moderately lower proportions of ProSPC^+^ and AQP5^+^ cells compared to H9 controls, while H1-derived organoids exhibited comparable but slightly reduced ProSPC^+^ and PDPN^+^ populations (Figure 1J, S2A-J), suggesting the need for further optimization of iPSC differentiation conditions.

To circumvent labor-intensive differentiation protocols, we systematically assessed hALOs' cryostability and functional recovery post-resuscitation. Brightfield imaging confirmed preserved organoid architecture following freeze-thaw cycles (Figure S3A). Quantitative analysis across passages (P3, P11, P28) demonstrated consistent cryopreservation resilience, with comparable expression levels of alveolar lineage markers (NKX2-1, SFTPC), proliferative activity (Ki67), and apoptosis markers (Cleaved-Caspase3) across all tested passages (Figure S3B-C).

Collectively, these findings establish hALOs' dual capacity for sustained expansion and repeated cryopreservation cycles without lineage drift or functional compromise. This technical advancement positions hALOs as a superior *in vitro* model for human alveolar physiology studies, particularly benefiting longitudinal experimental designs requiring stable cellular repositories.

### Single-cell profiling reveals developmental trajectories in hALOs

**Figure 2:**
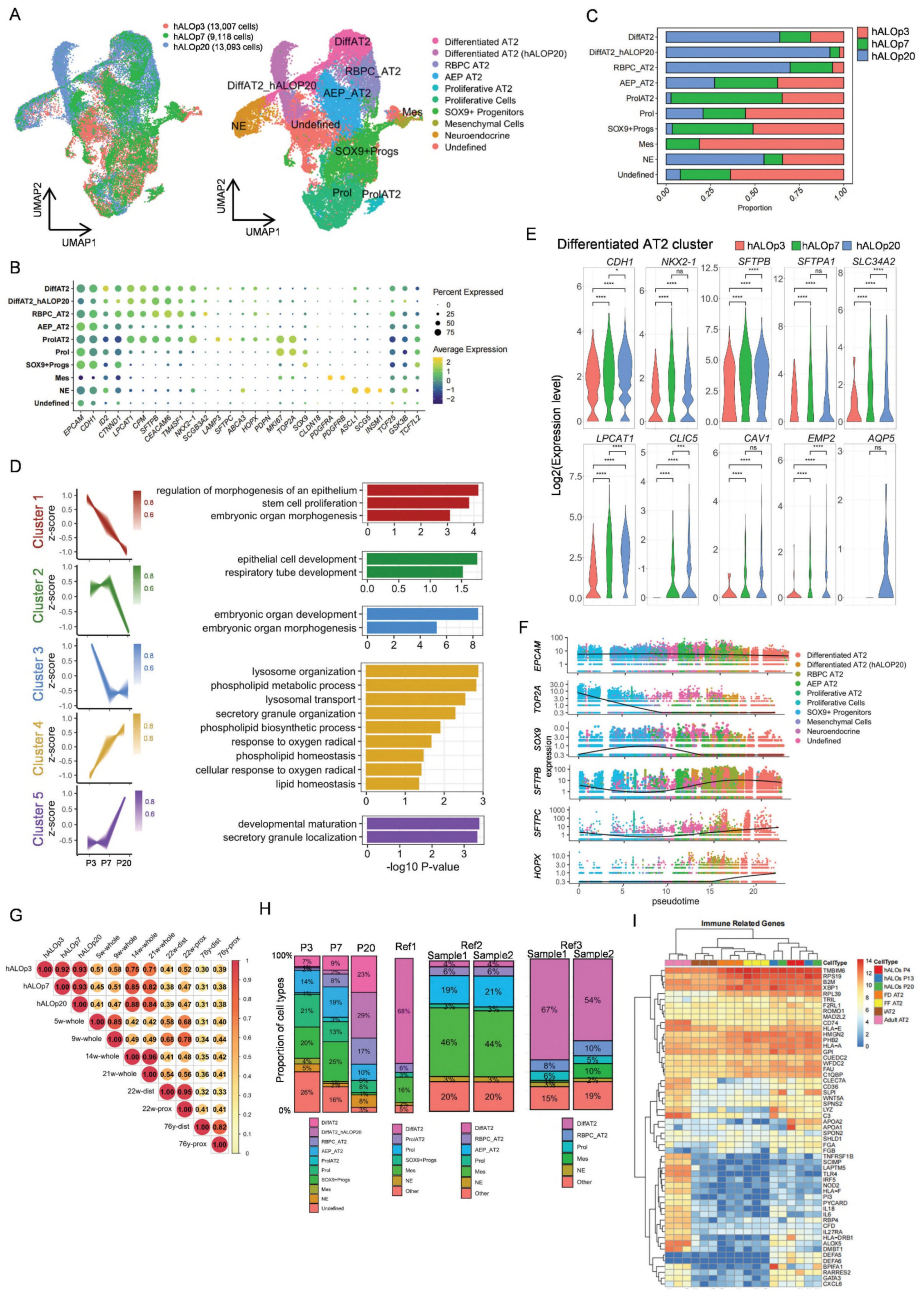
** Single-cell transcriptomic profiling of hALOs**. (A) Louvain clustering of the transcriptomes of hALOs. UMAP visualization illustrates the clustering of cell transcriptomes across different samples (left; red: hALOp3; green: hALOp7, blue: hALOp20) and identifies 10 distinct cell types (right; the abbreviated names are on the figure, and the full names are in the legend). (B) Expression of recognized marker genes in the dot plot of hALOs reflects the main components of hALOs. (C) Bar plot of hALOs' cell type proportions by sample. (D) Time course results of differentiated AT2 subpopulations derived from hALOs. The pseudobulk analysis identifies distinct gene expression trends (left) and provides a Gene Ontology analysis of biological processes for the genes enriched in these clusters (right). Clusters 1, 2, and 3 represent 3 distinct downstream trends (red: Cluster 1; green: Cluster 2; blue: Cluster 3), while Clusters 4 and 5 indicate 2 different upstream trends (yellow: Cluster 4; purple: Cluster 5). The shade of each color in the gene trend reflects the membership intensity of each gene within the corresponding cluster. (E) Violin plot depicting expression differences between AT1 and AT2 marker genes in the pseudobulk of the differentiated AT2 subcluster's hALOs (red: hALOp3; green: hALOp7, blue: hALOp20; * p<0.05, *** p<0.001, **** p<0.0001, ns not significant, Wilcoxon rank-sum test). (F) Gene expression trends along pseudotime for each subpopulation. (G) Heatmap of Pearson's correlation coefficients between hALOs and published fetal and adult lung data based on normalized expression of all genes across *EPCAM*^+^ cells (Details are provided in Figure S4). (H) Bar plot of cell type proportions in hALOs and three published hPSC-derived lung organoid systems (Ref1: GSE150708; Ref2: GSE162936; Ref3: GSE148113). (I) Heatmap of immune related genes in hALOs, iAT2s (FF/FD iAT2, SFTPC+ iAT2) and adult AT2.

To resolve the cellular composition and developmental progression of hALOs, we performed single-cell RNA sequencing (scRNA-seq) on organoids at early (P3), intermediate (P7), and late (P20) passages and identified ten distinct clusters (Figure 2A, B; S4A). The AT2 lineage comprised five subpopulations: differentiated AT2 cells (enriched with AT2 signals), P20-enriched AT2 subset showing upregulated organelle localization and protein secretion pathways (Figure S4B), respiratory bronchiole pulmonary cell-like AT2 (RBPC AT2, marked by *SCGB3A2*, *CEACAM6*, and select AT2 genes)^47^, alveolar epithelial progenitor-like AT2 (AEP AT2, featuring *TM4SF1*, WNT signaling genes *TCF25* and *TCF7L2*, along with lung developmental gene* ID2*)^55^, and proliferating AT2 cells (enriched with cycling and AT2 signatures). Additional populations included another cycling cells, SOX9^+^ progenitors, neuroendocrine cells (*ASCL1*^+^*SCG5*^+^), mesenchymal cells (*PDGFRA*^+^*PDGFRB*^+^), and a WNT-active undefined cluster.

Our data demonstrated dynamic cellular changes during culture, with AT2 populations expanding while progenitor clusters declined (Figure 2C). Pseudobulk analysis of the differentiated AT2 cells revealed coordinated upregulation of alveolar maturation pathways (lysosome organization, phospholipid metabolism) and downregulation of developmental genes involved in cell morphogenesis (Figure 2D). These molecular signatures were reinforced by the temporal expression patterns of AT2 markers (*SFTPB*, *SFTPA1*, *SLC34A2*, and* LPCAT1*), which showed significant increases from P3 to P7. Although scRNA-seq did not resolve a discrete AT1 cluster (possibly due to sampling constraints), we consistently detected AT1 transcripts (*CLIC5*, *CAV1*, *EMP2*, *AQP5*) within AT2 populations (Figure 2E), corroborating our qRT-PCR and immunofluorescence results.

Pseudotime analysis delineated a continuous progenitor to AT2 trajectory, characterized by the progressive loss of progenitor markers (*SOX9*) and concomitant activation of alveolar-specific genes (*SFTPB*, *SFTPC* and *HOPX*), recapitulating in vivo alveolar development^56^ (Figure 2F; S4C, D). Developmental benchmarking through integration with fetal lung datasets^47^, ^48^ positioned hALOs at the pseudoglandular-canalicular transition (14-21 weeks), faithfully recapitulating this critical stage of human lung maturation (Figure 2G; S4E-G). Importantly, the absence of contaminating airway (e.g. basal cells, ciliated cells, club cells, and goblet cells) or non-pulmonary cell types (e.g. liver, gut, thyroid, and forebrain) confirmed differentiation specificity (Figure S4H).

Together, these findings establish hALOs as a physiologically relevant model for studying human alveolar development and disease.

### Comparative transcriptomic profiling reveals cellular and functional retention in hALOs

To delineate cellular composition heterogeneity, we re-annotated our hALOs and three published hPSC-derived lung organoid systems (Ref1: GSE150708; Ref2: GSE162936; Ref3: GSE148113)^49^-^51^ using consistent cell-type definitions. Comparative analyses revealed distinct yet complementary cellular profiles across platforms. Notably, our system exhibited: (1) preservation of multiple AT2 subpopulations (including AEP, RBPC and proliferating AT2 subtypes), and (2) minimal mesenchymal lineage with absence of airway epithelial cells (Figure 2H, S4I). These findings demonstrate that differentiation platforms generate organoids with unique but overlapping cellular architectures, each offering specific advantages for modeling different aspects of alveolar biology.

While single-cell analyses revealed distinct cellular composition profiles across platforms, we next sought to evaluate the global transcriptomic fidelity of our system at the functional level. To comprehensively compare long-term cultured hALOs with primary human pulmonary cells and published organoid models, we performed bulk RNA-seq analyses against three reference cohorts from public databases: (1) PSC-derived pulmonary lineages, encompassing CPM antibody-isolated LPs (CPM^+^ LPs)^53^, H9 hESC-derived bud tip organoids^30^, induced respiratory airway progenitors (iRAPs)^52^, induced AT2 cells (iAT2s) generated under fibroblast-dependent (FD iAT2) or fibroblast-free (FF iAT2) conditions^12^, ^53^, and SFTPC^tdTomato^-sorted iAT2s^11^; (2) Human fetal lung specimens comprising uncultured tissue^30^, progenitor organoids^30^, and cultured 21-week gestation explants^11^; (3) Primary adult human AT2 cells^11^ (Figure S5A). We identified 2,000 highly variable genes across all samples, revealing transcriptional similarity between hALOs and iRAPs, likely attributable to shared culture conditions and non-enriched protocols (Figure S5B). Hierarchical clustering revealed robust alveolar marker expression profiles in hALOs, where surfactant-related genes (*SFTPB*, *SFTPA1*, *SFTPA2*) matched expression levels in purified iAT2s, while AT1 markers (*PDPN*, *HOPX*, *CAV1*) showed significantly higher expression than in iRAPs. This maturation signature was further supported by the downregulation of developmental regulators (*SOX9*, *FOXP2*, *FOXA2*) and GO analysis demonstrating enriched respiratory development, alveolar morphogenesis and gas exchange pathways in hALOs compared to iRAPs (Figure S5C-F).

Beyond their established roles as alveolar stem cells and surfactant producers maintaining pulmonary tension, AT2 cells possess critical immunomodulatory functions^57^. Comparative pathway analysis revealed that sorted iAT2s displayed reduced immune-response pathway activity compared to primary AT2 cells, consistent with previous reports of culture-induced attenuation of native AT2 immunoregulatory programs^58^ (Figure S5G). Notably, hALOs maintained higher expression levels of adult AT2-associated immune regulators (e.g., *HLA-DRB1*) and demonstrated greater enrichment for cytokine production and humoral immune response pathways relative to iAT2s (Figure 2I, S5G-I). These observations indicate that our culture system preserves certain aspects of innate immune functionality that might be compromised during cell isolation procedures.

### *In vitro* differentiation of hALOs by modulating WNT and YAP signaling

**Figure 3:**
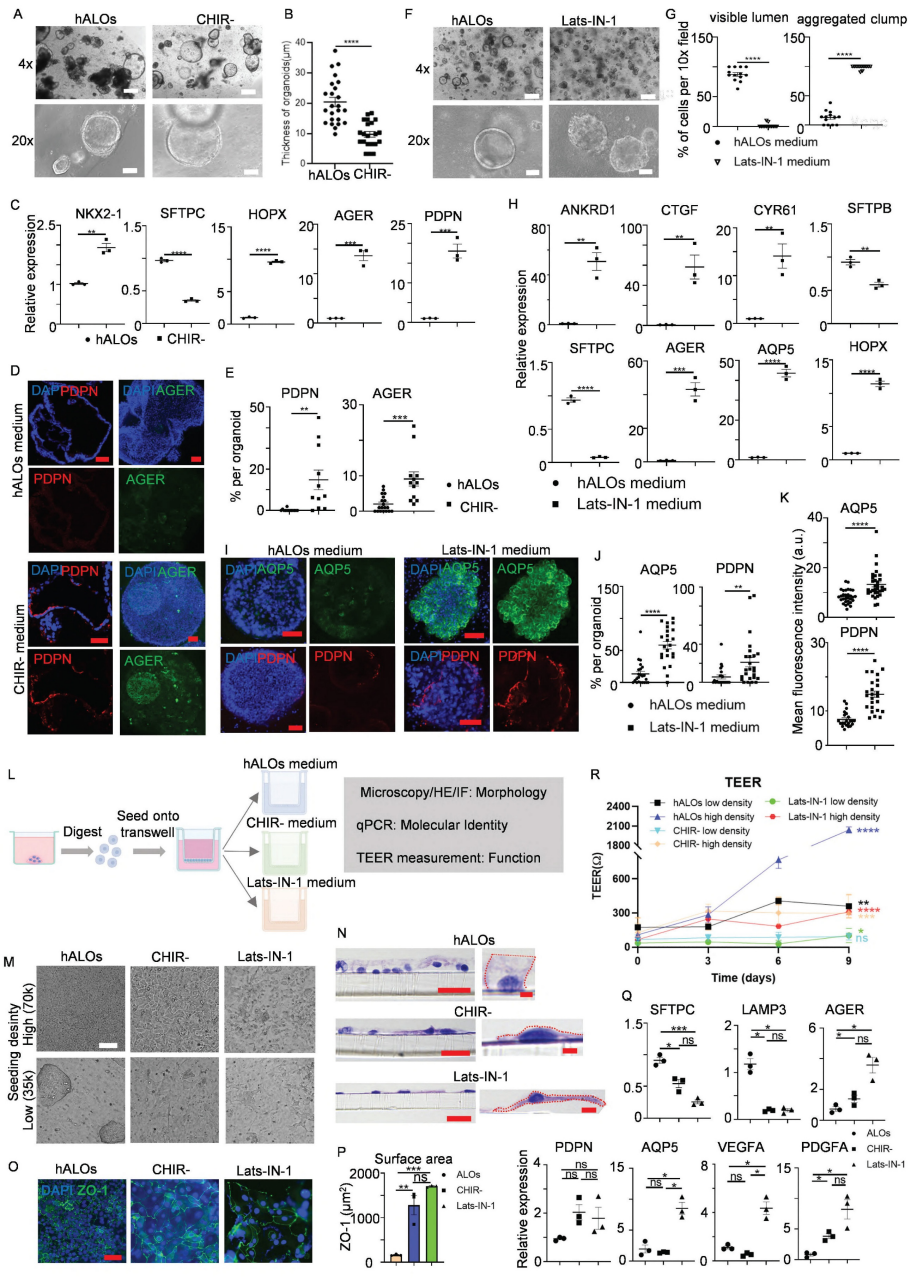
** Modulating WNT and YAP signaling enables functional AT1 cell generation in hALOs.** (A) Representative brightfield images of organoids in hALOs and CHIR- medium. 500 μm (top panel), 100 μm (bottom panel). (B) Quantification of the thickness of organoids in hALOs and CHIR- medium. **** p<0.0001 (unpaired, two-tailed Student's t-test). (C) mRNA expression level of lung lineage and alveolar related markers in hALOs and CHIR- medium. ** p<0.01, *** p<0.001, **** p<0.0001 (unpaired, two-tailed Student's t-test). (D, E) Representative immunofluorescence staining (D) and quantification (E) of AT1 marker PDPN (section staining) and AGER (whole-mount staining) in hALOs and CHIR- medium. ** p<0.01, *** p<0.001 (unpaired, two-tailed Student's t-test). Scale bars, 50 μm. (F, G) Representative brightfield images (F) and morphological quantification (G) of hALOs in control medium (hALOs medium) and Lats-IN-1 medium. **** p<0.0001 (unpaired, two-tailed Student's t-test). Scale bars, 500 μm (top panel), 100 μm (bottom panel). (H) mRNA expression level of YAP related gene and lung lineage markers in hALOs medium and Lats-IN-1 medium. ** p<0.01, *** p<0.001, **** p<0.0001 (unpaired, two-tailed Student's t-test). (I-K) Representative whole-mount immunofluorescence staining (I), quantification (J) and mean fluorescence intensity (K) of AT1marker AQP5 and PDPN in hALOs medium and Lats-IN-1 medium. ** p <0.01, **** p<0.0001 (unpaired, two-tailed Student's t-test). Scale bars, 50 μm. (L) Schematic of the experimental design. (M) Representative brightfield images of cells seeded at high (70k) and low (35k) densities and cultured under different conditions at the ALI for 9 days. Scale bars, 100 μm. (N) Representative H&E staining of cells seeded at low densities and cultured under different conditions at the ALI for 9 days. Scale bars, 20 μm (left panels); 2 μm (right panels in hALOs and CHIR-); 5μm (right panel in Lats-IN-1). (O) Representative immunofluorescence labeling for ZO-1 in cells seeded at low densities at the ALI for 9 days. Scale bars, 50 μm. (P) Quantification of cellular surface area based on ZO-1-defined cell boundaries in low-density cultures at the ALI after 9 days. **p<0.01, ***p<0.001 (one-way ANOVA; n=3 per condition). (Q) mRNA expression level of markers in different culture conditions at the ALI for 9 days. * p<0.05, *** p<0.001 (one-way ANOVA). (R) TEER of cells plated at high (70k) and low (35k) densities and cultured under different conditions at the ALI for 9 days. * p<0.05, ** p<0.01, *** p <0.001, **** p<0.0001 (unpaired two-tailed Student's t-tests were conducted to compare measurements at day 9 versus baseline (day 0) within each experimental condition).

Gas exchange, the primary alveolar function mediated by AT1 cells, remains challenging to study due to difficulties in isolating pure AT1 populations^59^. While previous studies suggest WNT signaling inhibition or agonist withdrawal enhances AT1 differentiation in multilineage organoids or fibroblast-dependent alveolar models^28^, ^29^, we investigated AT1 induction in our fibroblast-independent hALOs in early passages. CHIR99021 withdrawal (CHIR-) during days 30-37 eliminated exogenous WNT signaling, inducing flattened hALO morphology characteristic of AT1 generation versus CHIR+ controls^29^ (Figure 3A,B). qRT-PCR revealed marked upregulation of AT1 markers (*HOPX*, *AGER* and *PDPN*) concurrent with SFTPC downregulation under CHIR- conditions (Figure 3C). Immunofluorescence confirmed abundant PDPN^+^/AGER^+^ cells in CHIR--treated P2 hALOs versus near absence in CHIR+ cultures (Figure 3D, E). These coordinated molecular and phenotypic shifts demonstrate WNT pathway attenuation drives hALOs toward AT1-dominant states.

To enhance differentiation efficiency, we developed an optimized protocol based on established mechanisms of AT1 fate determination. Previous studies identified nuclear YAP/TAZ translocation as a conserved driver of AT1 differentiation^60^, ^61^. Besides, activation of TGF-β signaling and inhibition of FGF signaling could all bias lung progenitors toward an AT1 cell fate^62^. Building on these findings, we established a Lats-IN-1-dependent differentiation system (designated as Lats-IN-1 medium) by withdrawing CHIR99021/SB431542/KGF from the standard hALOs culture and adding Lats-IN-1 (LATS kinase inhibitor). This optimized condition induced luminal collapse and transformation into solid cellular aggregates within 9 days (Figure 3F, G). qRT-PCR analysis revealed coordinated upregulation of YAP transcriptional targets (*ANKRD1, CYR61* and *CTGF*) and AT1 specification markers (*AGER*, *AQP5* and* HOPX*), concurrent with suppression of AT2 identity genes (*SFTPB* and *SFTPC*) (Figure 3H). Whole-mount immunofluorescence staining indicated that AQP5- and PDPN-positive cells, as well as mean fluorescence intensity of each organoid, were enriched in Lats-IN-1 medium (Figure 3I-K). Cross-lineage validation using iPSC- and H1 hESC-derived hALOs demonstrated conserved AT1 induction efficacy, confirming robust pathway responsiveness (Figure S6A-E). These findings establish Hippo-LATS-YAP signaling as an evolutionarily conserved regulator of AT1 differentiation in human alveolar organoids.

To assess functional AT1 generation, we cultured dissociated organoids at high (70,000) and low (35,000) densities under three conditions: hALOs, CHIR-, and Lats-IN-1 media for 9-day ALI culture (Figure 3L). Bright-field and H&E analysis showed hALOs maintained cuboidal AT2 morphology, forming clusters at low density, while CHIR- and Lats-IN-1 conditions produced flattened, elongated AT1-like cells (Figure 3M, N). ZO-1 staining revealed significantly larger cell areas in CHIR- and Lats-IN-1 versus hALOs (Figure 3O, P). Molecular analysis showed AT2 gene (*SFTPC*, *LAMP3*) downregulation and AT1 marker upregulation (*AGER*, *AQP5*), most pronounced in Lats-IN-1 cultures. Furthermore, Lats-IN-1 medium specifically induced expression of signaling ligands *PDGFA* and *VEGFA*, recapitulating the known paracrine signaling function of AT1 cells in vivo^63^ (Figure 3Q).

Functional assessment of barrier properties by TEER measurements revealed density-dependent differences. Under high-density conditions, all culture groups showed significant increases in electrical resistance compared to baseline measurements, with hALOs cultures exhibiting the highest resistance values, followed by CHIR- and Lats-IN-1 conditions. This pattern is consistent with previous reports comparing AT2 and AT1 barrier properties^60^. In low-density cultures, only hALOs-maintained cells showed substantial resistance increases, while Lats-IN-1 cultures displayed minimal improvement and CHIR- cultures showed no significant change, indicating impaired barrier formation in AT1-dominant cultures at lower cell densities (Figure 3R).

These results demonstrate successful AT1 generation through molecular, morphological and functional characterization, providing a robust platform for studying human AT1 biology.

### *In vivo* long-term transplantation and differentiation of hALOs

**Figure 4:**
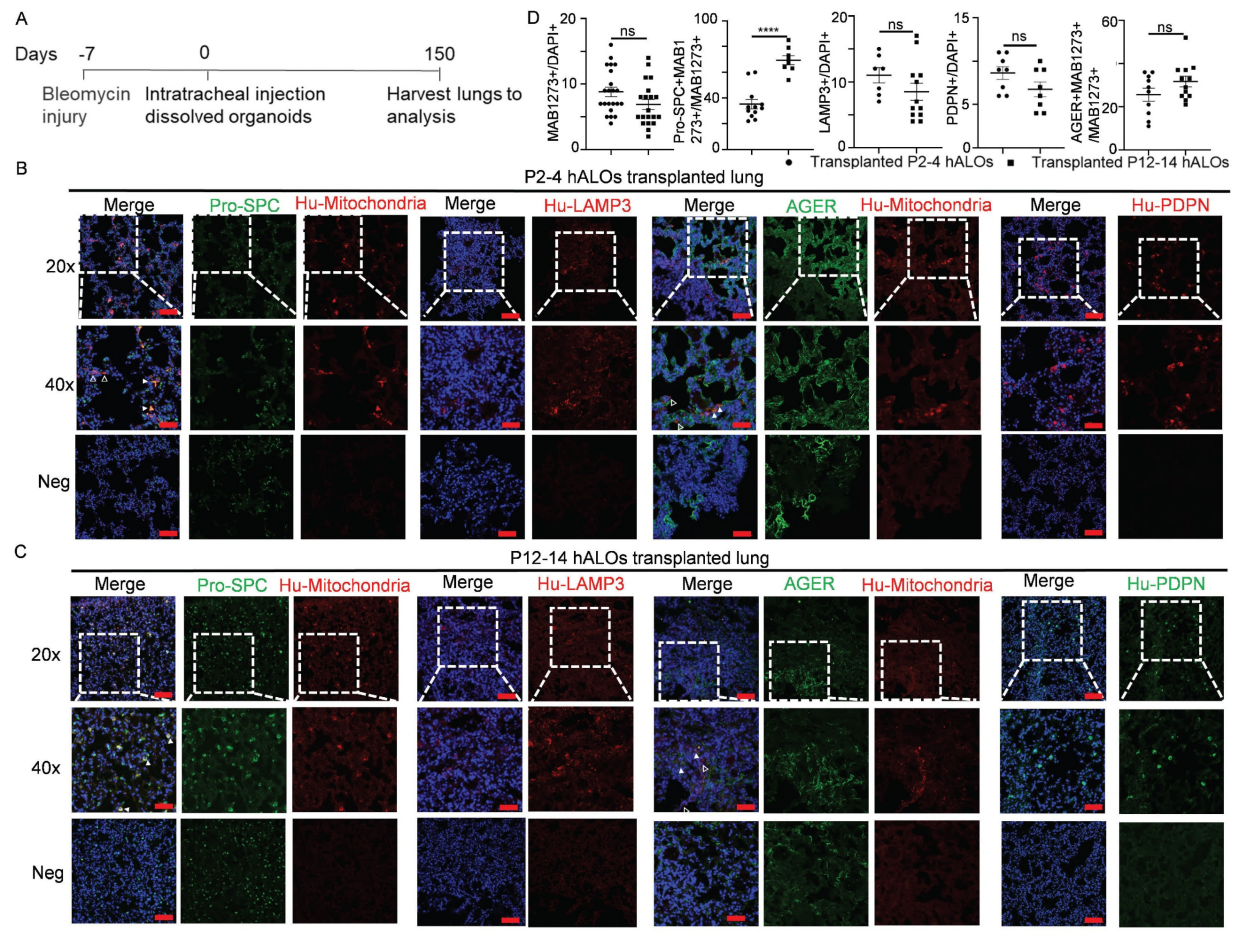
***In vivo* long-term engraftment and differentiation of hALOs.** (A) Schematic of the experimental design. (B-D) Representative immunofluorescence labeling and quantification (D) of human mitochondria marker MAB1273, AT2 marker pro-SPC and LAMP3, as well as AT1 marker human PDPN (HU-PDPN) and AGER in transplanted P2-4 (B) and P12-14 (C) hALOs. The regions without human mitochondria or human PDPN expression are shown as negative control. n=7 mice (P2-4), n=8 mice (P12-14). Scale bars, 100 μm (top and bottom panels in B and C); 50 μm (middle panels in B and C).

To evaluate *in vivo* translational potential, we assessed engraftment capacity and lineage differentiation of hALOs following xenotransplantation into bleomycin-injured lungs of immunodeficient B-NDG mice (Figure 4A). Both early (P2-P4) and late passage (P12-P14) hALOs demonstrated persistent survival, with human mitochondrial marker MAB1273+ cells detected at 8.82% ± 0.72% and 6.90% ± 0.75% engraftment efficiencies, respectively (Figure 4B-D). Engrafted populations exhibited progressive alveolar maturation: MAB1273+ cells demonstrated AT2 commitment through co-localization with pro-SPC (35.25% ± 3.60% in P2-P4 vs 69.38% ± 3.44% in P12-P14), while exclusively expressing human LAMP3 in 11.00% ± 1.15% (P2-P4) versus 8.50% ± 1.30% (P12-P14) of cells. Concurrently, AT1 differentiation was evidenced by MAB1273/AGER co-localization (25.60% ± 3.00% vs 31.67% ± 2.49%) and autonomous human PDPN expression (8.63% ± 0.73% vs 6.75% ± 0.84%) (Figure 4B-D). These findings establish hALOs' capacity for long-term in vivo survival and functional alveolar lineage progression, underscoring their utility for modeling pulmonary regeneration mechanisms.

### DNA delivery of hALOs

To establish an hALOs system capable of modeling human development and disease, comprehensive characterization of its genetic modification capabilities is essential. We systematically evaluated three distinct transfection methodologies—Polyethylenimine (PEI) transfection, electroporation, and lentiviral infection—using a CAG-mNeonGreen expression vector across all approaches (Figure S7A). For PEI and electroporation protocols, PiggyBac (PB) transposon was co-delivered to facilitate stable genomic integration. Flow cytometric analysis revealed transfection efficiencies of 2.70% ± 1.05% for PEI, 3.92% ± 0.71% for electroporation, and 21.3% ± 4.71% for lentiviral vectors (Figure S7B). Lentiviral vectors demonstrated superior efficiency, establishing optimal parameters for stable genome manipulation.

We further assessed transfection efficiency across hALOs derived from diverse PSC sources by introducing Lenti-EF1α-mCherry into hALOs generated from H9 hESCs, H1 hESCs, and iPSCs. All examined cell lines exhibited robust transduction efficiencies: 71.1%, 67.5%, and 60.4% respectively (Figure S7C). This consistency underscores the method's broad applicability across PSC lineages. Notably, the observed superior performance of Lenti-EF1α-mCherry compared to Lenti-CAG-mNeonGreen may stem from differences in vector size, as larger constructs often exhibit reduced transduction efficiency in lentiviral systems.

### CRISPR-Cas-mediated genetic manipulation of hALOs

CRISPR-Cas technology had been rapidly adopted in stem cells and tissue-derived organoids^64^-^66^, but rarely in hPSC-derived lung organoids. Capitalizing on our established transfection capacity, we implemented three CRISPR-based strategies in hALOs: transcriptional activation, gene knock-in and knock-out engineering.

**Figure 5:**
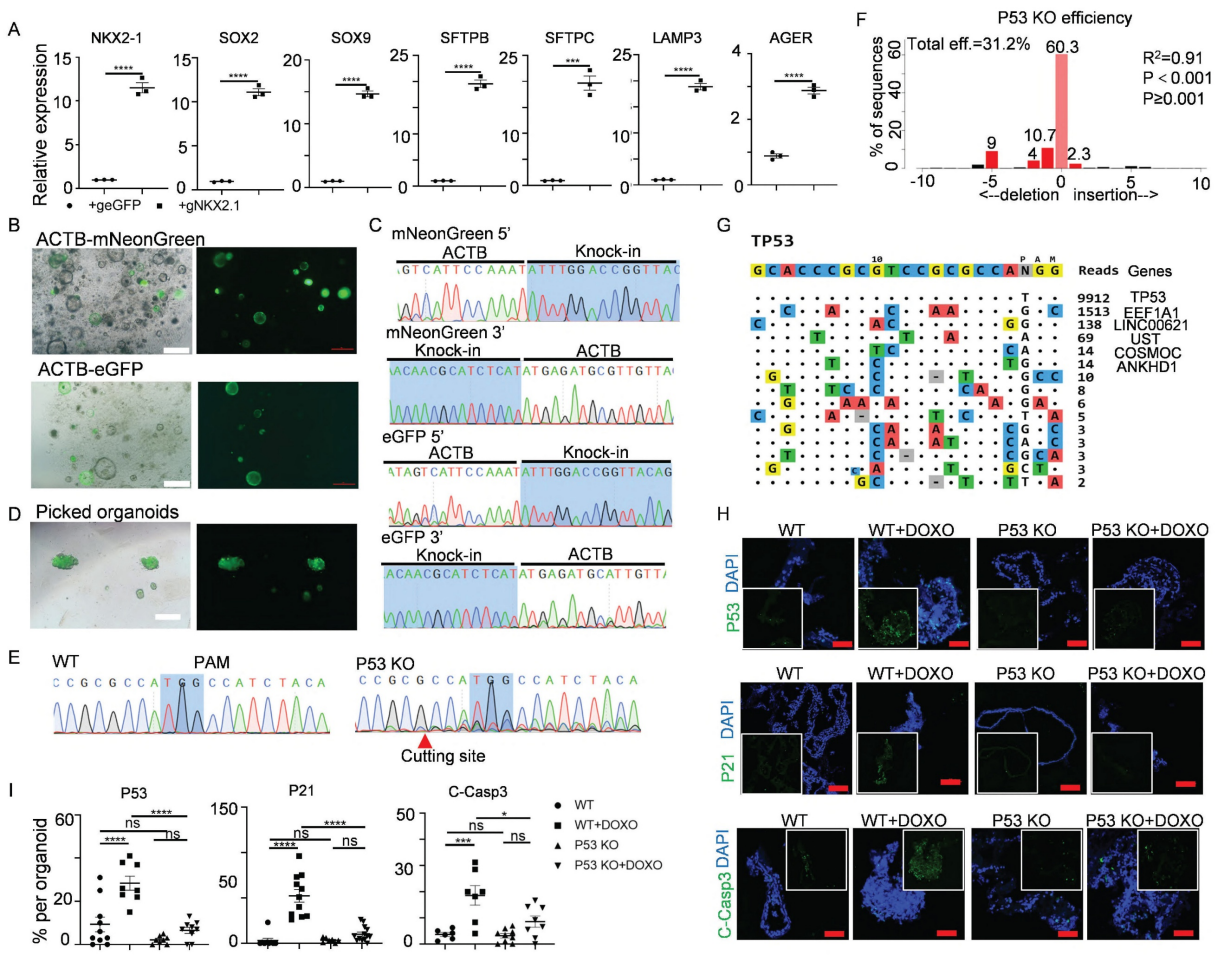
** Gene activation, knock-in and knock-out in hALOs.** (A) mRNA levels of differentiation markers in Cas9-P300 expressing hALOs after transfection of *eGFP* gRNA control and *NKX2.1* gRNA. *** p<0.001, **** p<0.0001 (unpaired, two-tailed Student's t-test). (B) Representative brightfield and fluorescence images of hALOs with* mNeonGreen* or *eGFP* gene knocked-in at the *ACTB* site. Scale bars, 500 μm. (C) Sequencing of the 5′ and 3′ junction sites at the knock-in allele. (D) Representative brightfield and fluorescence images of picked fluorescence-positive organoids. Scale bars, 500 μm. (E) Sequencing of WT and organoids transfected with *TP53* gRNA. The protospacer adjacent motif (PAM) and cutting site are indicated. (F) Knock-out efficiency was analyzed by TIDE. (G) Tag-seq profiling the off-target sites induced by Cas9 with *TP53* sgRNA. (H, I) Representative immunofluorescence staining (H) and quantification (I) of P53, P21 and Cleaved-Caspase3 in WT and P53 knockout organoids treated with or without 1μM doxorubicin. * p<0.05, *** p <0.001, **** p<0.0001 (one-way ANOVA). Scale bars, 50 μm (P53 and Cleaved-Caspase3 staining), 100 μm (P21 staining).

For targeted gene activation, we leveraged a doxycycline (Dox)-inducible Cas9-P300 transcriptional activation system (iKA-CRISPR)^33^ to modulate *NKX2-1* - a master regulator of alveolar fate specification through chromatin remodeling^67^. Delivery of truncated gRNAs (14-15 bp targeting the *NKX2-1* promoter) into iKA-CRISPR hALOs induced over 10-fold *NKX2-1* upregulation with concomitant increases in LPs/AT2/AT1 markers compared to eGFP-gRNA controls (Figure 5A).

To enable precise knock-in modifications, we employed a Cas9-SB100X fusion protein (Cas9-N57) that synergizes CRISPR targeting with transposase-mediated DNA integration^68^. Brightfield, fluorescence images and sequencing of the PCR amplicons showed that we successfully knocked *mNeonGreen* or *eGFP* into the β-actin (*ACTB*) locus (Figure 5B, C). Furthermore, fluorescence-positive organoids could be picked out to generate a knock-in organoid line (Figure 5D).

We next evaluated CRISPR-Cas9-mediated knockout feasibility using *TP53*, a tumor suppressor governing genomic stability and carcinogenesis^69^, ^70^. Transfection of sgRNAs targeting *TP53* into iKA-CRISPR hALOs achieved 31.2% knockout efficiency via TIDE analysis^71^, with Sanger sequencing confirming frameshift mutations (Figure 5E,F). To address off-target concerns, Tag-seq genome-wide profiling^34^ revealed 81.2% on-target reads (*TP53* locus) versus <18.8% at predicted off-target sites (*EEF1A1*, *LINC00621*, *UST*) (Figure 5G), with subsequent Sanger sequencing confirming intact off-target loci (Figure S8), demonstrating robust on-target specificity.

To assess functional consequences, we challenged wild-type (WT) and TP53-KO hALOs with doxorubicin (1 μM, 48h) - a DNA damage agent activating TP53-dependent apoptosis^72^, ^73^. Immunofluorescence revealed that the expression of *TP53*, *TP53* target gene *P21*, and apoptosis marker Cleaved-Caspase 3 were significantly upregulated in WT hALOs treated with doxorubicin, while the upregulation of these genes in P53 KO hALOs was minor (Figure 5H, I). These data indicated that the functional knock-out of *TP53* was achieved in hALOs.

Taken together, these multiplexed genetic manipulations establish hALOs as a versatile platform for lineage tracing, fate mapping, and functional genomics in human alveolar biology.

### Modeling of early-stage lung adenocarcinomas (LUAD) upon *KRAS^G12D^* expression in hALOs

**Figure 6:**
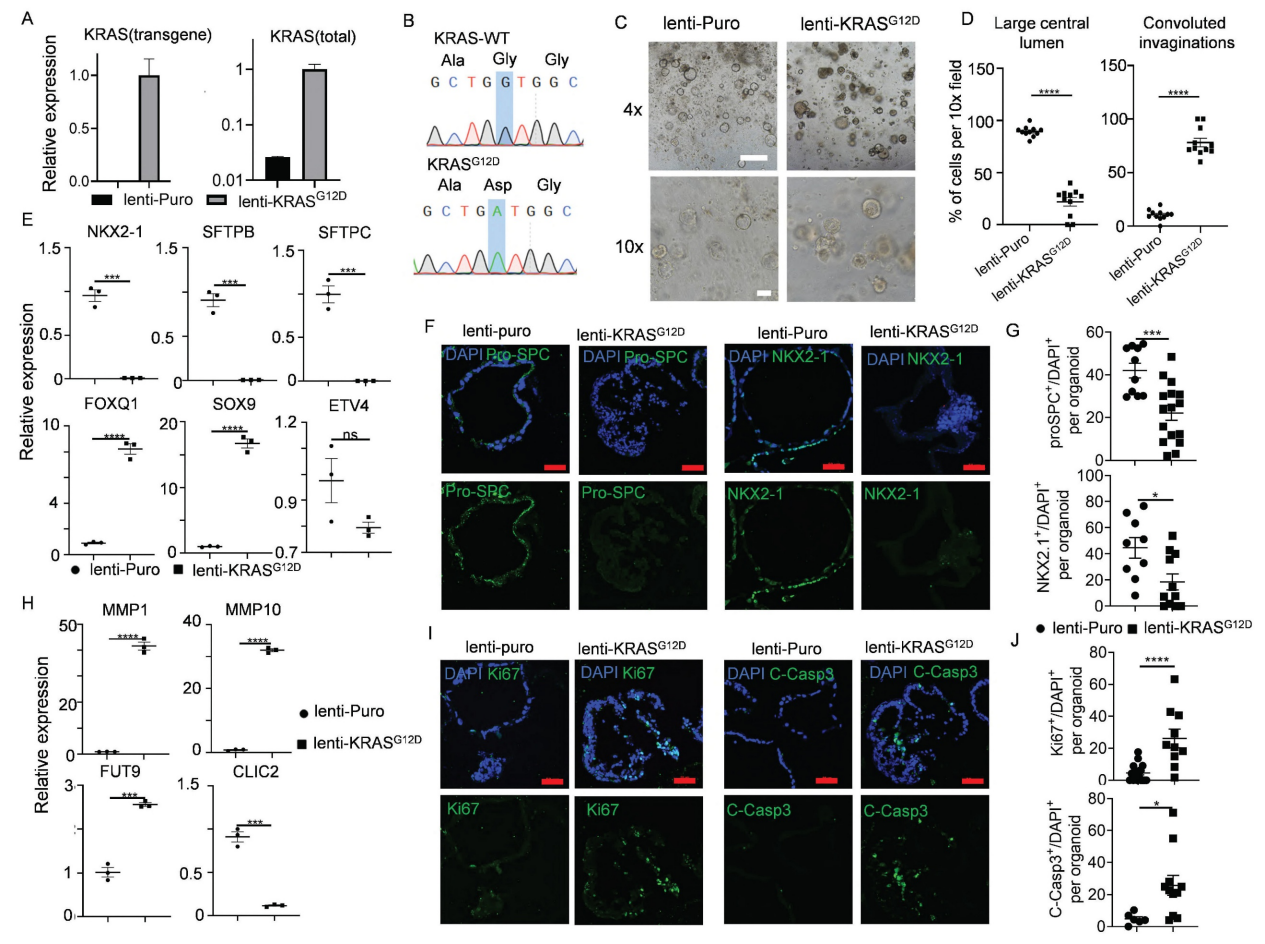
** Early-stage lung adenocarcinomas modeling upon *KRAS^G12D^* expression in hALOs.** (A, B) *KRAS^G12D^* mutation was identified by qRT-PCR and Sanger sequencing. (C, D) 10 days after infection with lentivirus, representative brightfield images (C) and morphological quantification (D) of the control group (lenti-puro) and lenti-KRAS^G12D^ group. **** p<0.0001 (unpaired, two-tailed Student's t-test). Scale bars, 500 μm (top panel), 100 μm (bottom panel). (E) mRNA levels of the lung epithelial markers in the control group (lenti-puro) and lenti-KRAS^G12D^ group. *** p<0.001, **** p<0.0001 (unpaired, two-tailed Student's t-test). (F, G) Representative immunofluorescence staining (F) and quantification (G) of lung lineage marker NKX2.1 and AT2 marker pro-SPC in control group (lenti-puro) and lenti-KRAS^G12D^ group. * p<0.05, *** p<0.001 (unpaired, two-tailed Student's t-test). Scale bars, 50 μm. (H) mRNA levels of the extracellular matrix markers and metabolic markers in the control group (lenti-puro) and lenti-KRAS^G12D^ group. *** p<0.001, **** p<0.0001 (unpaired, two-tailed Student's t-test). (I, J) Representative immunofluorescence staining (I) and quantification (J) of proliferation marker Ki67 and apoptosis marker Cleaved-Caspase3 in the control group (lenti-puro) and lenti-KRAS^G12D^ groups. * p<0.05, **** p<0.0001 (unpaired, two-tailed Student's t-test). Scale bars, 50 μm.

Oncogenic KRAS mutation is a powerful driver for various cancers and exists in nearly 30% of LUAD^74^. Certain early events of non-small cell lung cancer (NSCLC), such as cellular morphological alterations, metabolic/proliferative dysregulation, and dedifferentiation phenomena, could be modeled using organoids derived from *KRAS^G12D^* mutated PSCs^31^, ^75^. We asked whether this mutation could be achieved directly in hALOs. Successful integration was confirmed by KRAS^G12D^-specific transcript detection (Figure 6A) and Sanger sequencing (Figure 6B). Striking morphological shifts were observed: control organoids maintained characteristic multicystic architecture, whereas KRAS^G12D^-hALOs developed solid morphology with convoluted invaginations (Figure 6C, D). qRT-PCR and immunofluorescence revealed concomitant downregulation of alveolar lineage markers (*NKX2-1*, *SFTPB*, *SFTPC*) (Figure 6E-G) alongside upregulated progenitor signatures (*FOXQ1*, *SOX9*) (Figure 6E). Metabolic dysregulation manifested through elevated extracellular matrix remodeling factors (MMP1, MMP10, FUT9) and suppressed glutathione pathway components (CLIC2) (Figure 6H), recapitulating reported premalignant metabolic shifts^31^. *KRAS* mutated hALOs exhibited concurrent elevation of proliferative (Ki67^+^) and apoptotic (Cleaved-Caspase3^+^) indices (Figure 6I, J), mirroring the paradoxical growth dynamics of early tumorigenesis.

These findings demonstrate that direct genomic perturbation of hALOs recapitulates KRAS-driven premalignant transformation events, establishing their utility for interrogating lung adenocarcinoma initiation mechanisms.

### Orthotopic transplantation of KRAS^G12D^-mutated hALOs induces LUAD-like phenotype

**Figure 7:**
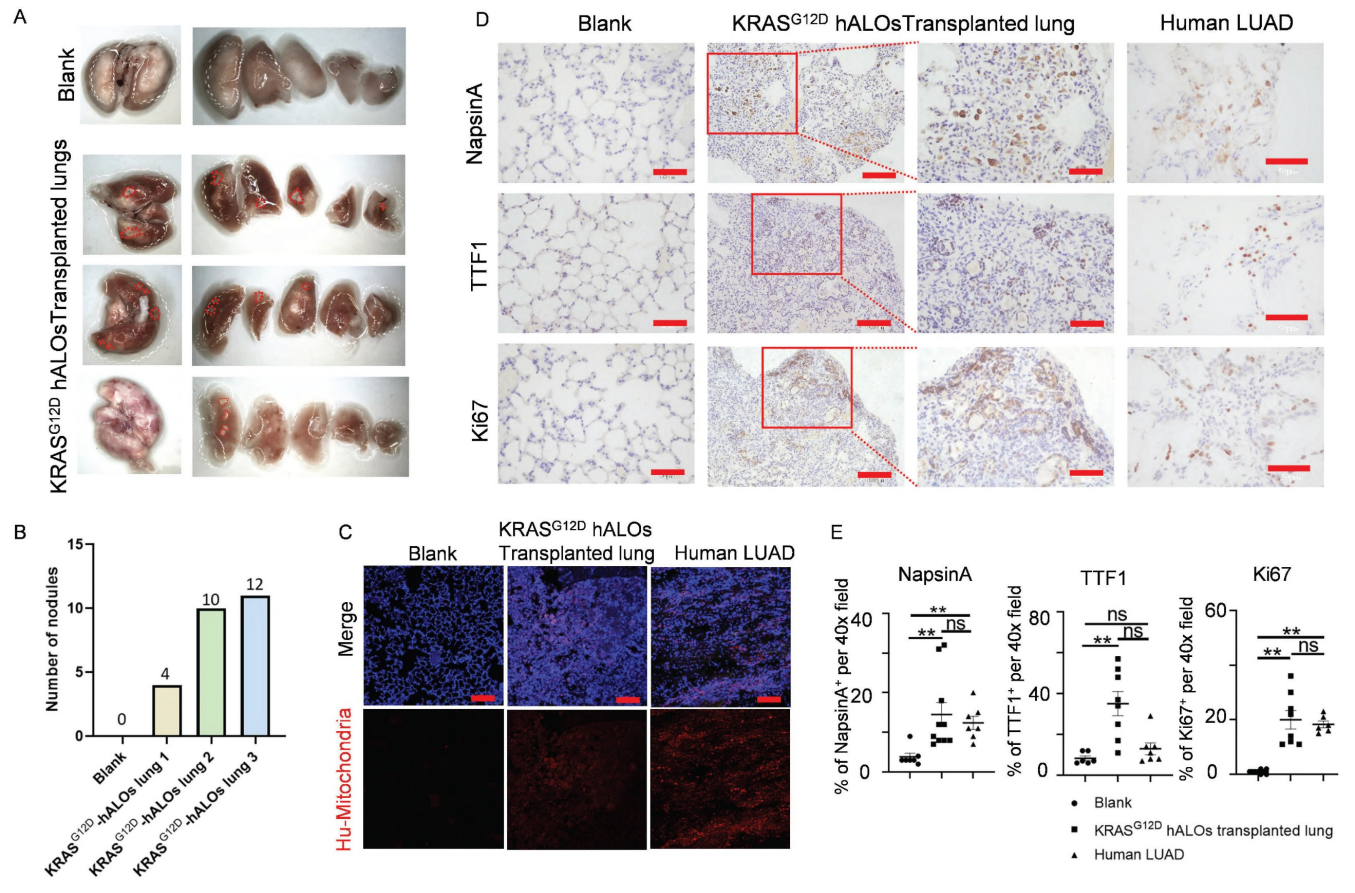
** Orthotopic transplantation of KRAS^G12D^-mutated hALOs induced LUAD-like phenotype.** (A) Representative photos of mouse lung of blank control or transplanted with KRAS^G12D^-mutated hALO (n=3 mice). (B) Quantification of the number of nodules in (A). (C) Representative immunofluorescence labeling of human mitochondria marker MAB1273 in blank, KRAS^G12D^-mutated hALOs transplanted lung and human LUAD sample. Scale bars, 100 μm. (D) Representative immunohistochemistry images of samples staining for proliferation marker Ki67 and LUAD marker NapsinA and TTF1. 100 μm (left panels in KRAS^G12D^-mutated hALOs transplanted lung); 50 μm (Blank, human LUAD and right panels in KRAS^G12D^-mutated hALOs transplanted lung). (E) Quantification of immunohistochemistry staining positive cells per 40× field in each sample. ** p<0.01 (Kruskal-Wallis tests followed by Dunn's post hoc tests with Bonferroni correction).

To evaluate malignant transformation potential, we performed orthotopic xenotransplantation of KRAS^G12D^-hALOs into bleomycin-injured B-NDG mice, representing the first demonstration of *in vivo* pulmonary carcinogenesis modeling using hPSC-derived alveolar organoids. The cohort achieved 50% long-term survival (≥150 days; 3/6 mice) with macroscopic tumor nodule formation (Figure 7A, B), contrasting with acute mortality (3/6) in the first fortnight attributable to infection-related complications without tumorigenic lesions. Immunofluorescence showed that clonal human mitochondrial-positive cells were formed in KRAS^G12D^ hALOs transplanted lung, while normal hALOs grafts were scattered (Figure 7C; 4B, C). Immunohistochemistry detected multiple regions positive for LUAD markers NapsinA and TTF1, as well as proliferation marker Ki67 in the lungs of KRAS^G12D^ hALOs transplanted mice (Figure 7D, E; S9A). However, no region was detected positive for lung squamous cell carcinoma (LSCC) markers P40 and CK5/6, indicating that KRAS mutant organoid transplantation can induce a phenotype resembling LUAD (Figure S9B-D).

## Discussion

In this study, we developed an hPSC-derived ALOs system, exhibiting robust long-term expansion, efficient cryopreservation and resuscitation capabilities, alongside potential for further differentiation and genetic modification. This system enabled us to successfully recapitulate early events of KRAS^G12D^-driven tumorigenesis and achieve orthotopic lung transplantation in mice. Critically, hALOs recapitulate alveolar epithelial dynamics with human-specific fidelity, overcoming the physiological limitations of transformed lines like A549 that lack authentic surfactant expression and polarized architecture^76^.

The hALO system addresses two critical gaps in pulmonary research: First, unlike ASC-derived organoids constrained by limited proliferative capacity^77^, hPSC-derived hALOs permit longitudinal studies of alveolar homeostasis and injury repair through near-unlimited expansion. Second, our systematic evaluation confirmed preserved ultrastructure (including lamellar body integrity and tight junction networks) and maintained functionality (surfactant secretion, immune-regulatory signatures, barrier function) following expansion/cryopreservation, while achieving industrial-scale reproducibility comparable to immortalized cell lines but without their tumorigenic potential. Furthermore, the elimination of fibroblast dependence and cell sorting requirements minimizes batch variability, enabling standardized disease modeling.

While current efforts focus primarily on transplantable LPs^78^, ^79^ or purified AT2 cells^12^, ^32^, physiologically relevant alveolar models preserving native cellular diversity remain scarce. Our hALOs provide a complementary approach with several notable features: (1) scRNA-seq analysis demonstrates a well-defined cellular composition, maintaining key alveolar lineages while minimizing off-target cell types, yet retaining rare but biologically relevant populations such as RBPC AT2 and AEP AT2 cells; (2) Unlike sorted populations, they preserve native AT2 immunoregulatory signatures (e.g., HLA-DRB1); (3) Longitudinal analysis shows progressive maturation from progenitor-like to distal lung phenotypes during passaging. Together, these characteristics - including alveolar lineage fidelity, experimental tractability (supporting long-term culture and genetic manipulation), and maintained multifunctionality - position hALOs as a valuable system for investigating alveolar development, differentiation and immune microenvironment interactions. However, hALOs may be less suited than purified AT2 cultures for investigating cell-autonomous mutation effects (e.g., *SFTPB^121ins2^*, *SFTPC^I73T^*). The functional roles of identified AT2 subpopulations also require validation. Future studies should develop standardized methods to isolate and characterize these subsets, improving disease modeling precision while preserving the system's strength in maintaining native cellular interactions.

The inherent immaturity of hPSC-derived hALOs, predominantly comprising lung progenitors and AT2 cells, poses significant challenges for modeling AT1-mediated physiology^21^, ^26^. Current strategies, primarily modulating WNT signaling, have shown mixed outcomes due to variations in methods and culture conditions^11^, ^28^, ^29^. Our study revealed that withdrawal of the WNT agonist CHIR99021 accelerated early AT1 differentiation, suggesting enhanced differentiation process. Building on recent findings^60^, ^80^, we also inhibited LATS kinases in hALOs, harnessing Hippo-LATS-YAP signaling essential for AT1 specification, and observed a marked increase in AT1 lineage. Concurrently, we withdrew inhibitors of AT1 differentiation like WNT, KGF, and TGF-β signals^62^, leading to organoid morphological shifts with underlying mechanisms yet to be elucidated. Furthermore, leveraging mechanical tension, a known* in vivo* maintainer of AT1 identity^59^, as a potential approach to enhance AT1 differentiation *in vitro* is promising. Through multidimensional engineering spanning molecular, microenvironmental, and systems-level optimization^21^, hALOs are evolving into high-fidelity alveolar proxies capable of deconstructing AT2-AT1 transition dynamics, screening lineage-specific therapeutics, and identifying fate-determining nodes. However, the precise mechanistic interplay between WNT inhibition and YAP activation in driving AT1 differentiation remains to be fully elucidated, particularly regarding their potential synergistic effects on alveolar epithelial fate determination.

CRISPR-based genome editing in lung organoids has predominantly focused on PSC-stage modifications to model developmental disorders (SFTPB deficiency, Hermansky-Pudlak syndrome)^11^, ^81^ and chronic pathologies (IPF, LUAD)^75^, ^82^, ^83^. However, early genetic perturbations risk compromising differentiation fidelity or introducing confounding pleiotropic effects^62^. Our demonstration of direct hALO editing—encompassing transcriptional activation, knockin, and knockout—circumvents these limitations while enabling precise functional interrogation of alveolar-specific processes. This post-differentiation engineering paradigm preserves epithelial integrity, offering a transformative toolset for investigating acquired lung diseases without developmental bias.

Notably, somatic KRAS^G12D^ introduction in mature hALOs—contrasting prior germline-like PSC engineering^31^—recapitulated LUAD-specific transformation (NapsinA^+^/TTF1^+^ nodules) without squamous features, mirroring mutation acquisition timing in human carcinogenesis^82^. Despite initiating premalignant lesions (prolonged survival >5 months, stable weight), KRAS^G12D^ alone proved insufficient for full malignancy, corroborating the multistep nature of lung adenocarcinoma progression^72^. This phenotypic bottleneck underscores the necessity of cooperative genetic hits—particularly tumor suppressor inactivation—to breach oncogenic checkpoints. Future combinatorial editing in hALOs could systematically map cooperativity networks driving malignant transition, bridging the gap between initiating mutations and clinical tumorigenesis.

Looking ahead, the continued advancement of co-culture and co-differentiation techniques, combined with advanced platforms such as microfluidics and organ-on-a-chip systems, will enable the integration of endothelial, immune, and fibroblast cells into lung organoid models^84^. These innovations will create more physiologically relevant systems that better mimic in vivo organs, representing an important inspiration for our future research. In parallel, while our data demonstrate long-term mitochondrial signals in transplanted murine lungs, we recognize that mitochondrial transfer may contribute to tissue repair independent of sustained engraftment, as reported in other stem cell systems^85^, ^86^. This suggests mitochondrial transfer alone could mediate lung repair. Future investigations will examine the potential protective effects mediated by hALO-derived mitochondria independent of cellular engraftment.

## Supplementary Material

Supplementary figures and tables.
