# Supplementary material for: Human Pluripotent Stem Cell-Derived Alveolar Organoids for Gene-editing and Lung Adenocarcinomas Modeling
Source: Int J Biol Sci. 2025 Oct 1;21(14):6285–304. doi: 10.7150/ijbs.118304 (PMC12594594; doi:10.7150/ijbs.118304)
Supplement: Supplementary file 1 — Supplementary figures and tables. [file ijbsv21p6285s1.pdf]

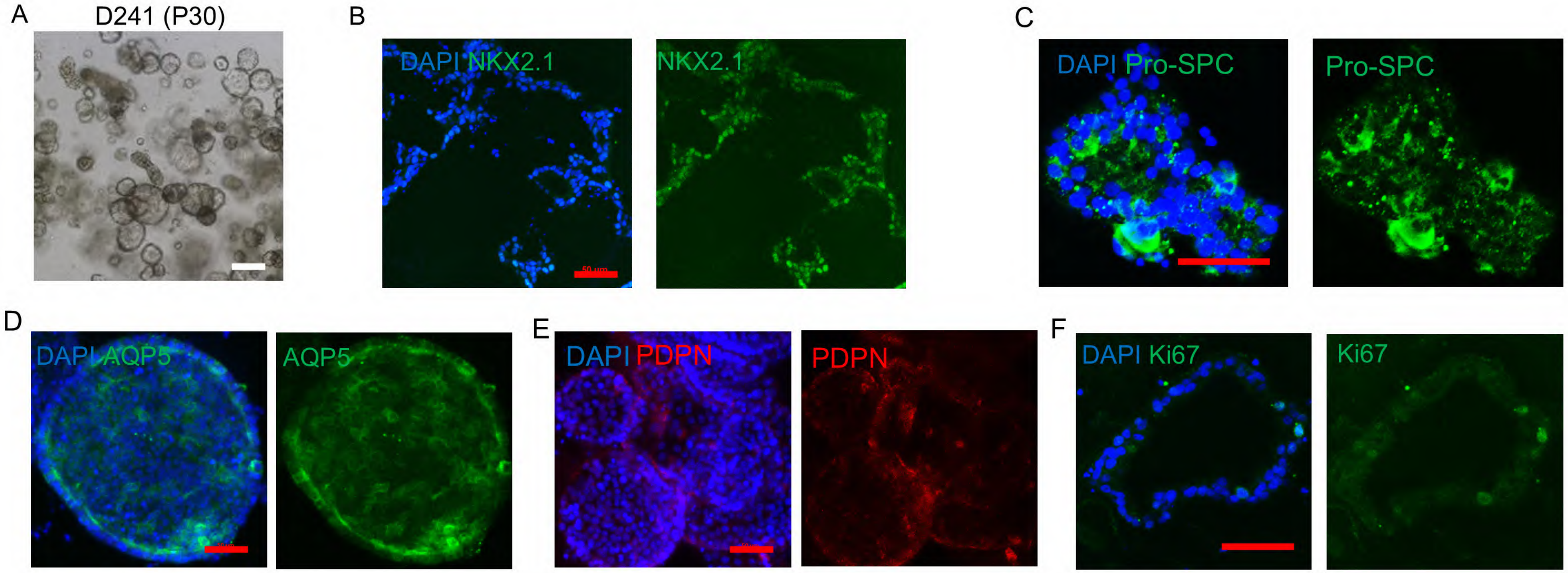

Figure S1 Characterization of long-term expanded hALOs.

Figure S1. Characterization of long-term expanded hALOs. (A) Representative brightfield images of hALOs at day241 (P30). Scale bars, 500 μm. (B-F) Representative immunofluorescence labeling of hALO markers at P30. Scale bars, 50 μm.

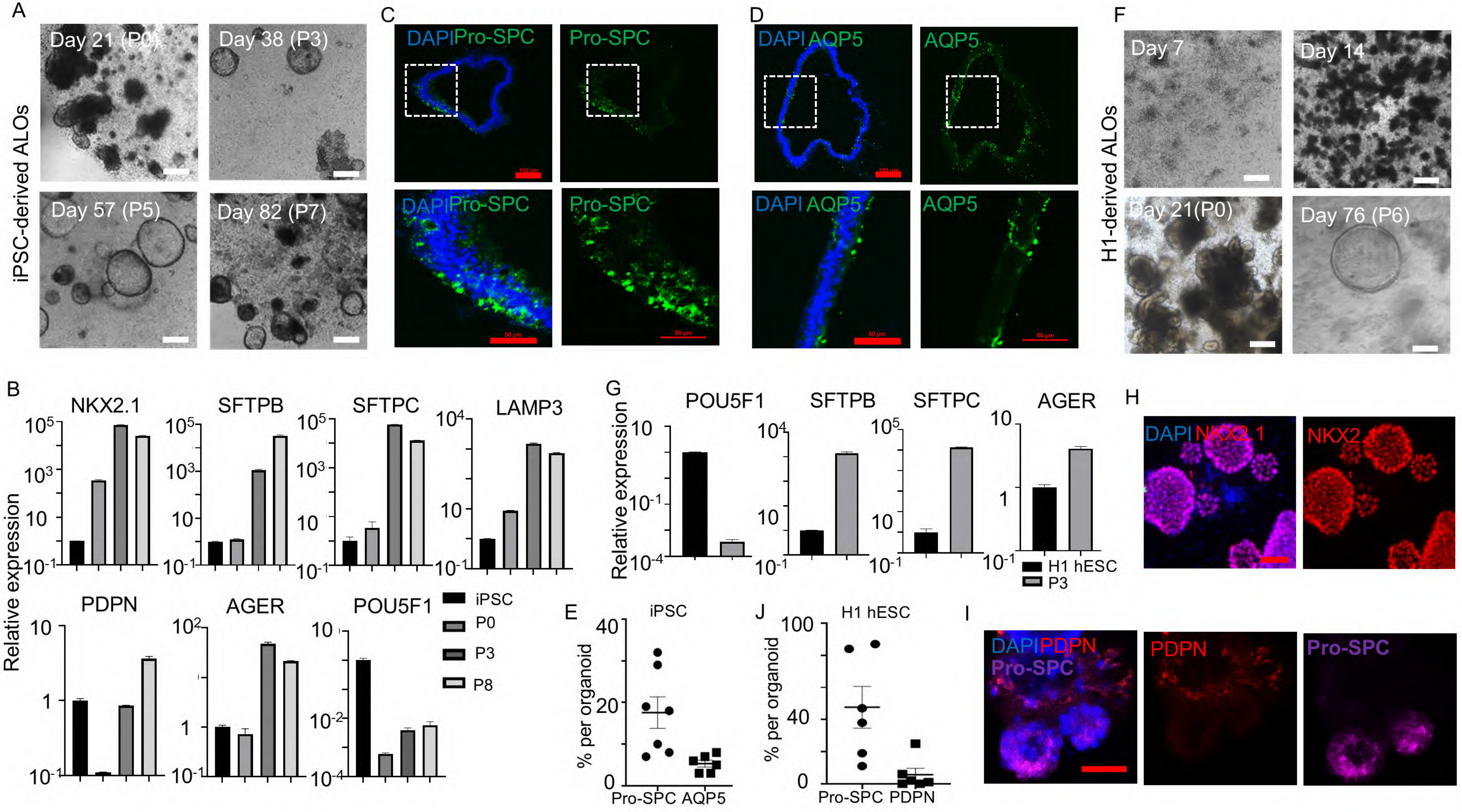

Figure S2 hALOs derived from hiPSC and H1 hESC.

Figure S2. hALOs derived from hiPSCs and H1 hESCs. (A) Representative brightfield images of iPSC derived hALOs at day21 (P0), day38 (P3), day 57 (P5) and day 82(P7). Scale bars, 500 μm. (B) mRNA expression levels of differentiation markers in hiPSCs-derived hALOs. (C-E) Representative immunofluorescence labeling (C, D) and quantification (E) of differentiation markers in hiPSCs-derived hALOs. Scale bars, 100 μm (top panel), 50 μm (bottom panel). (F) Representative brightfield images of H1 hESCs-derived hALOs at day7, day14, day21(P0) and day76 (P6). Scale bars, 500 μm. (G) mRNA expression levels of differentiation markers in H1 derived hALOs. (H-J) Representative immunofluorescence labeling (H, I) and quantification (J) of differentiation markers in H1-derived hALOs. Scale bars, 50 μm.

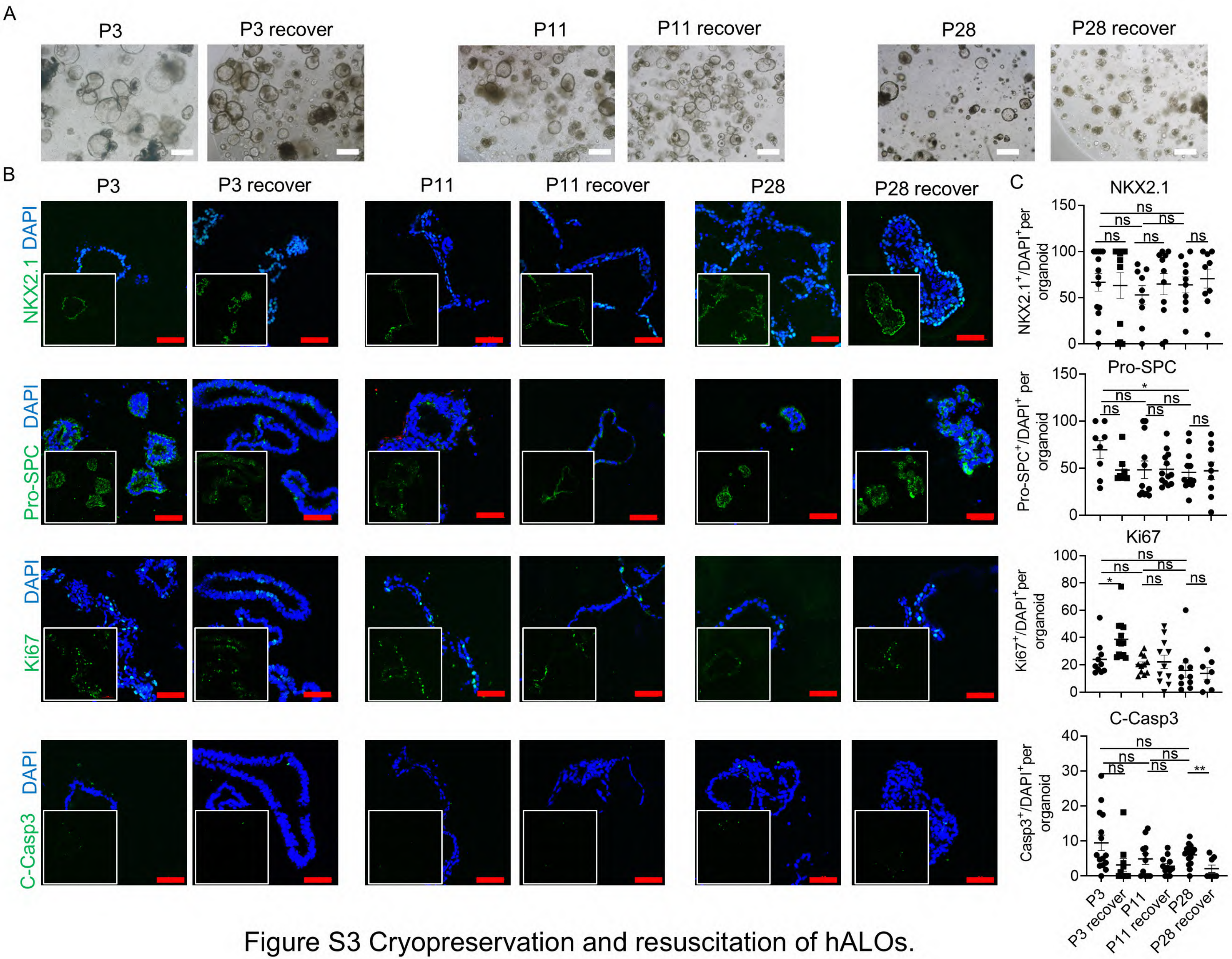

Figure S3 Cryopreservation and resuscitation of hALOs.

Figure S3. Cryopreservation and resuscitation of hALOs. (A) Representative brightfield images of hALOs prior and after freezing at different passage. Scale bars, 500 μm. (B-C) Representative immunofluorescence labeling (B) and quantification (C) of markers prior and after freezing at different passage. * $p < 0.05$, ** $p < 0.01$ (unpaired, two-tailed Student's t test). Scale bars, 50 μm.

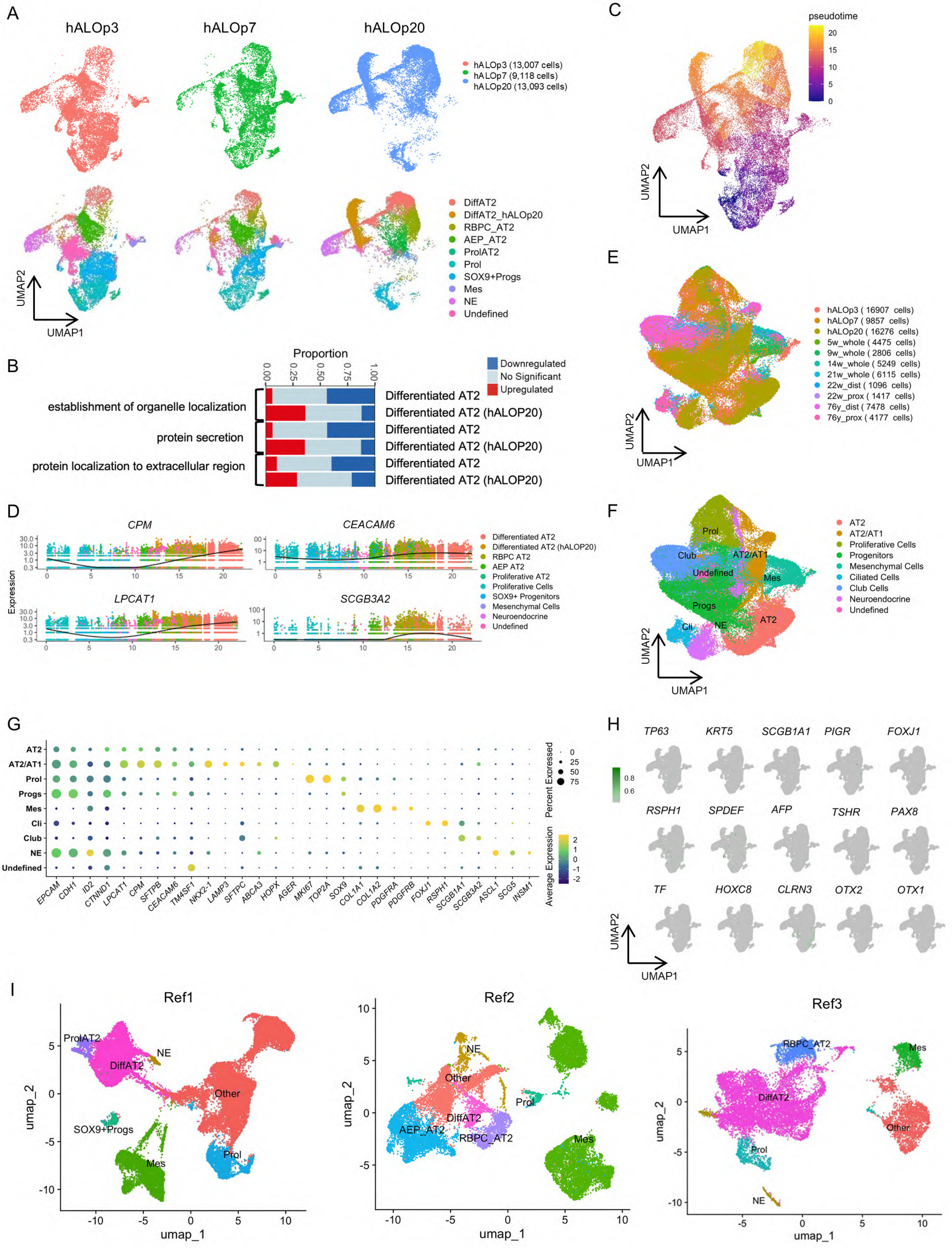

Figure S4. Single-cell transcriptomic profiling and integrated analysis of hALOs and cells from public databases.

Figure S4. Single-cell transcriptomic profiling and integrated analysis of hALOs and cells from public databases. (A) Louvain clustering of hALOs' cell transcriptomes. UMAP visualization shows the clustering of cell transcriptomes across different samples (top; red: hALOP3; green: hALOP7, blue: hALOP20) and the distinct cell types identified (bottom). (B) The proportions of genes related to maturation biological processes that are upregulated (red), downregulated (blue), and not significantly different (gray) in the differentiated AT2 subpopulation and differentiated AT2 (hALOP20) subpopulation. (C) Monocle3 prediction of the developmental trajectory of the EPCAM+ subpopulation of hALOs according to pseudotime. From purple to yellow indicates the progression of pseudotime from early to late.(D) Expression of recognized marker genes in addition to the primary genes shown in the trends along pseudotime. (E, F) Louvain clustering of hALOs and developmental stage cells. UMAP visualization displays the clustering of cell transcriptomes across different samples (E) and the identified distinct cell types (F). (G) Expression of recognized marker genes in dot plot reflects the primary components of the combined samples. (H) UMAP visualization demonstrating expression of established marker genes for: basal cells (*TP63*, *KRT5*), club cells (*SCGB1A1*, *PIGR*), ciliated cells (*FOXJ1*, *RSPH1*), goblet cells (*SPDEF*), liver cells (*AFP*, *TF*), gut cells (*HOXC8*, *CLRN3*), thyroid cells (*PAX8*, *TSHR*), and forebrain cells (*OTX1*, *OTX2*). (I) UMAP visualization illustrates the clustering of cell transcriptomes across different samples (Ref1: GSE150708; Ref2: GSE162936; Ref3: GSE148113).

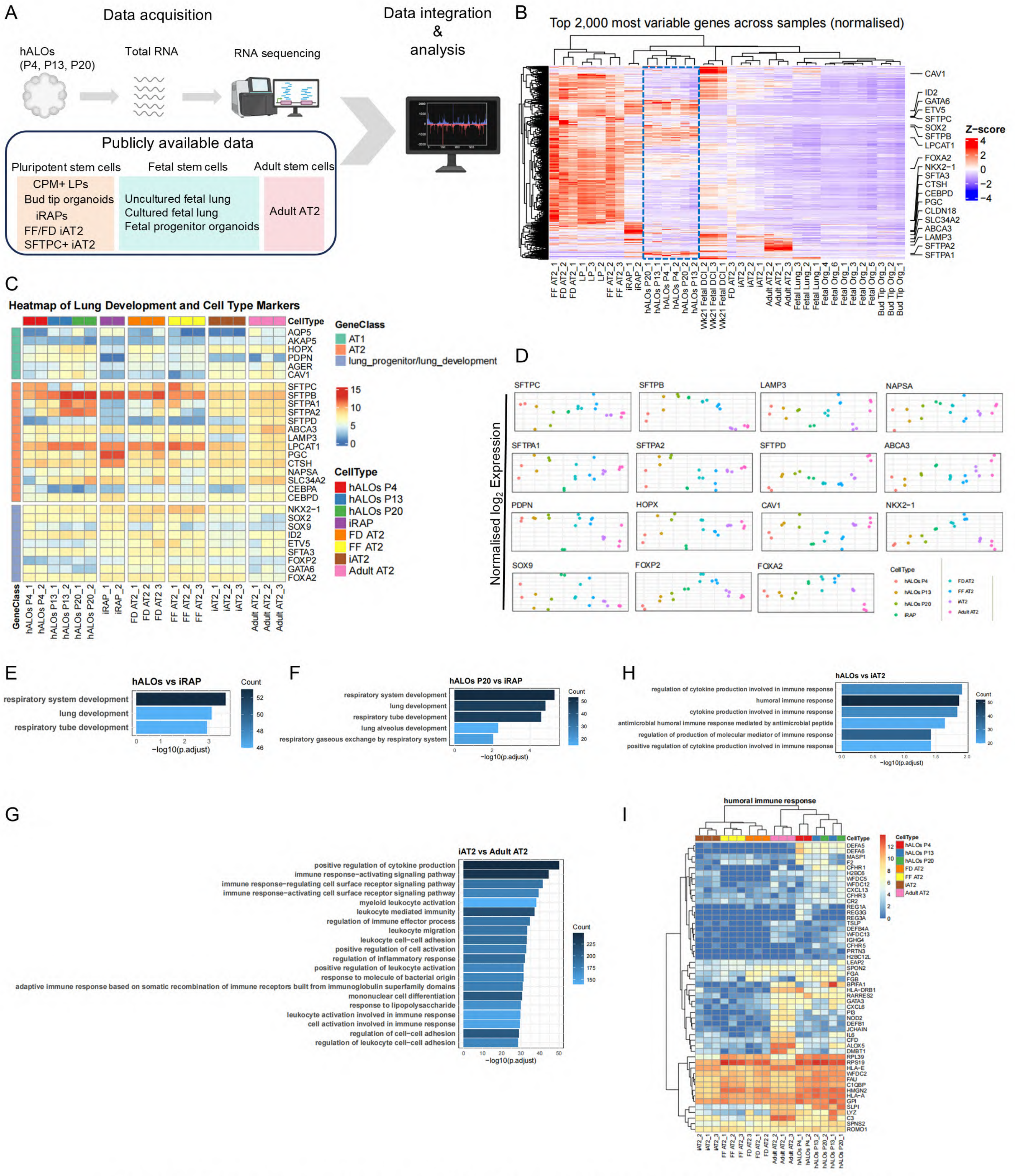

Figure S5 Transcriptomic comparison of hALOs with primary and engineered alveolar models

Figure S5 Transcriptomic comparison of hALOs with primary and engineered alveolar models. (A) Schematic overview of the acquisition, integration and analysis of RNA-sequencing data. (B) Unsupervised clustering heatmap displaying expression profiles of the top 2,000 most variable genes across all samples. (C) Heatmap of lung developmental regulators (progenitor/AT2/AT1-associated genes) in hALOs, iRAPs, iAT2s (FF/FD iAT2, SFTPC+ iAT2) and adult AT2. (D) Expression of key lineage specification markers in hALOs, iRAPs, iAT2s (FF/FD iAT2, SFTPC+ iAT2) and adult AT2. (E) GO pathway enrichment in hALOs (P4/P13/P20) versus iRAPs. (F) GO pathway enrichment in P20 hALOs versus iRAPs. (G) Top 20 depleted pathways in iAT2s (FF/FD iAT2, SFTPC+ iAT2) compared to adult AT2. (H) GO analysis of signaling pathways enriched in hALOs (P4, P13, P20) compared iAT2s (FF/FD iAT2, SFTPC+ iAT2). (I) Heatmap of gene set associate with humoral immune response in hALOs, iAT2s (FF/FD iAT2, SFTPC+ iAT2) and adult AT2.

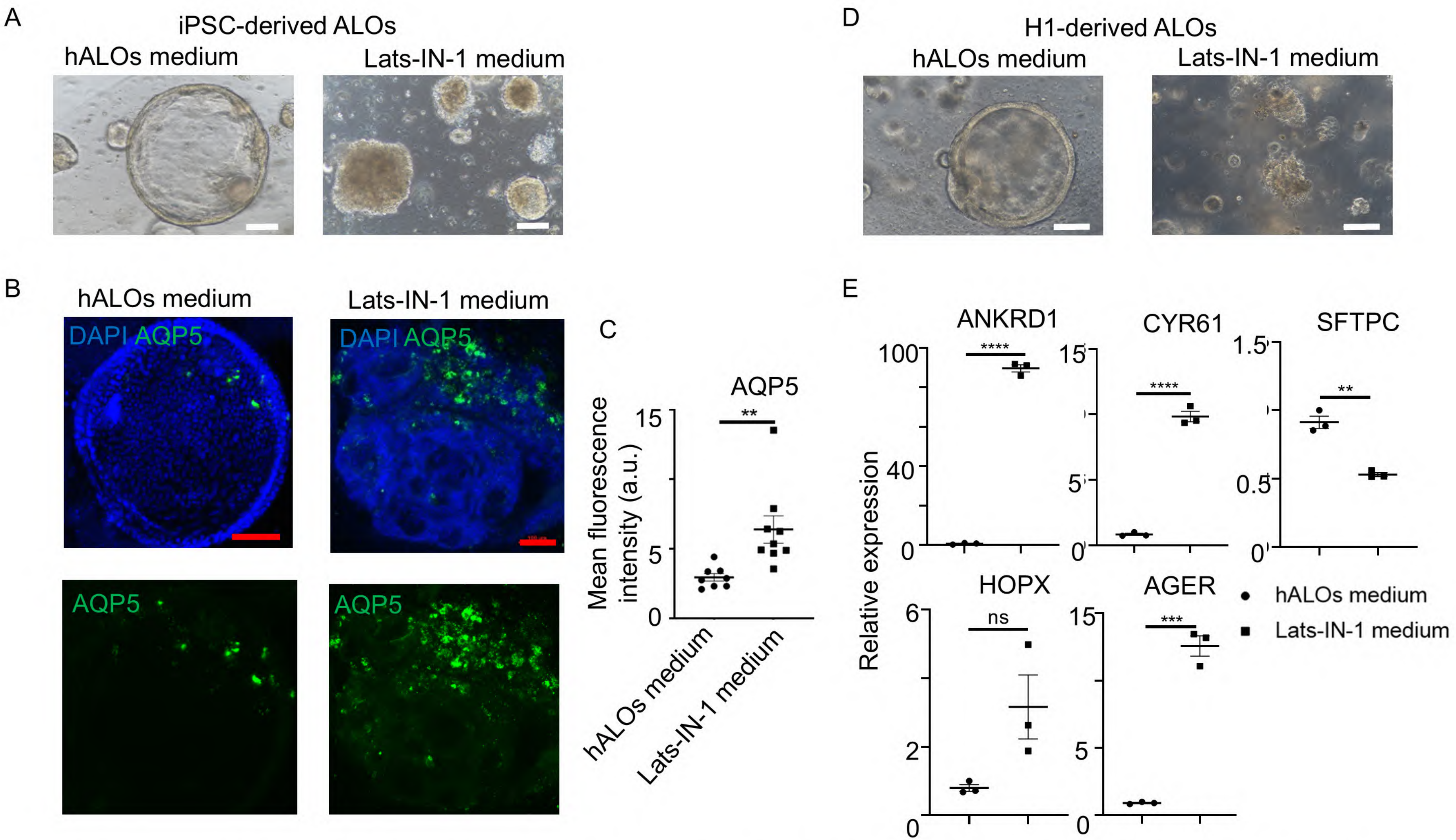

Figure S6 The activation of YAP signaling promotes the differentiation of iPSC and H1 derived ALOs.

Figure S6. The activation of YAP signaling promotes the maturation of iPSC and H1 derived ALOs. (A) Representative brightfield images of iPSC-derived hALOs in control medium (hALOs medium) and Lats-IN-1 medium. Scale bars, 500 μm. (B, C) Whole-mount immunofluorescence staining (B) and mean fluorescence intensity (C) of AT1 marker AQP5 in iPSC-derived hALOs. ** $p<0.01$ (unpaired, two-tailed Student's t test). Scale bars, 100 μm. (D) Representative brightfield images of H1 ESC-derived hALOs in control medium (hALOs medium) and Lats-IN-1 medium. Scale bars, 500 μm. (E) mRNA expression level of H1 ESC-derived hALOs. Similar results were observed in three independent biological replicates of RT-qPCR, and data from one representative replicate are shown. ** $p <0.01$, *** $p <0.001$, **** $p<0.0001$ (unpaired, two-tailed Student's t test).

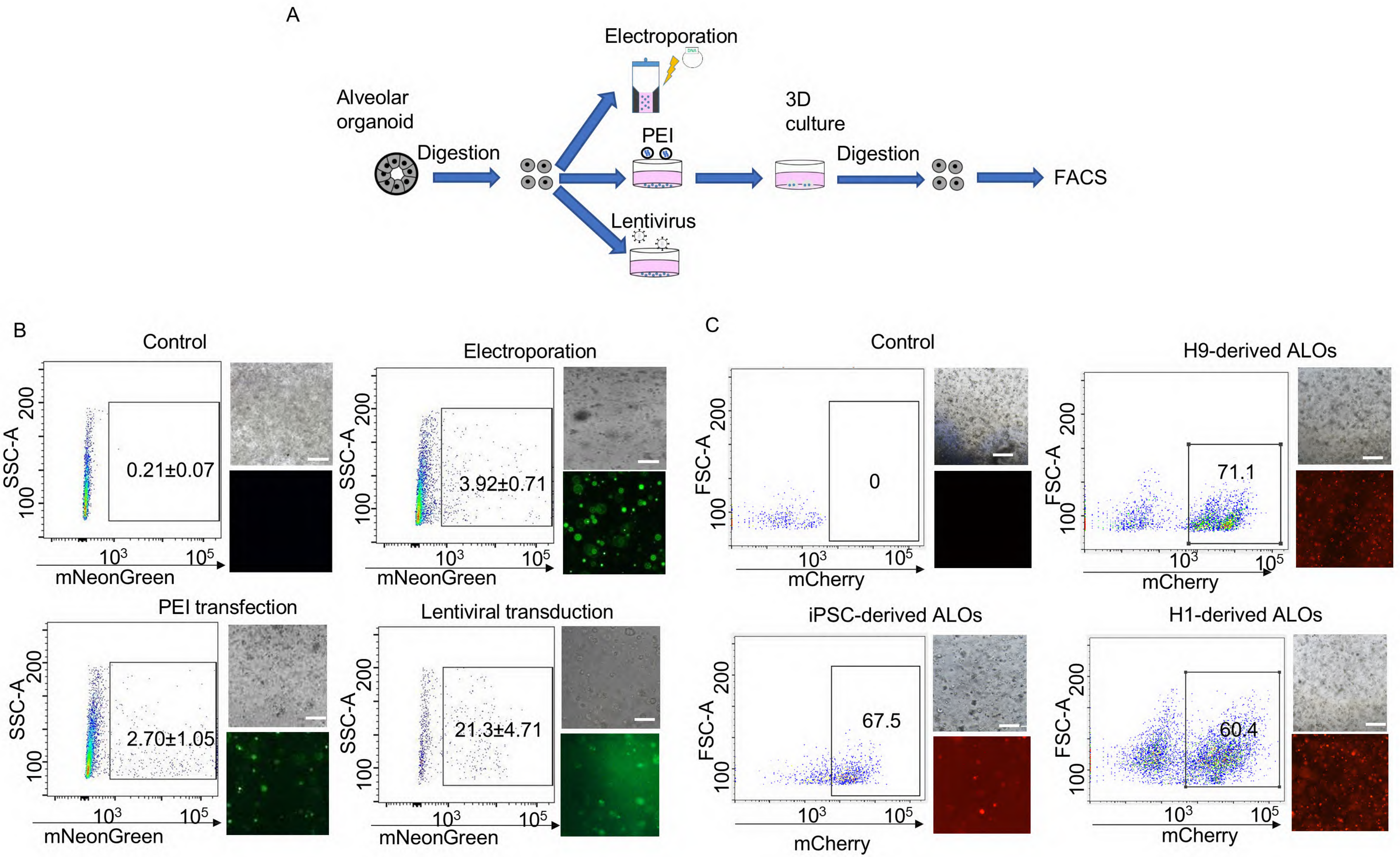

Figure S7 DNA delivery of hALOs.

Figure S7. DNA delivery of hALOs. (A) Schematic representation of the protocol of gene delivery of hALOs. (B) Representative flow-cytometry analysis showing different efficiency of electroporation, PEI transfection and lentivirus transduction. Data represent mean ± SEM (n=3). Representative brightfield and fluorescence images were shown. Scale bars, 500 μm. (C) Efficiency identification of lenti-mCherry transfection in H9, iPSC and H1-derived hALOs. Scale bars, 500 μm.

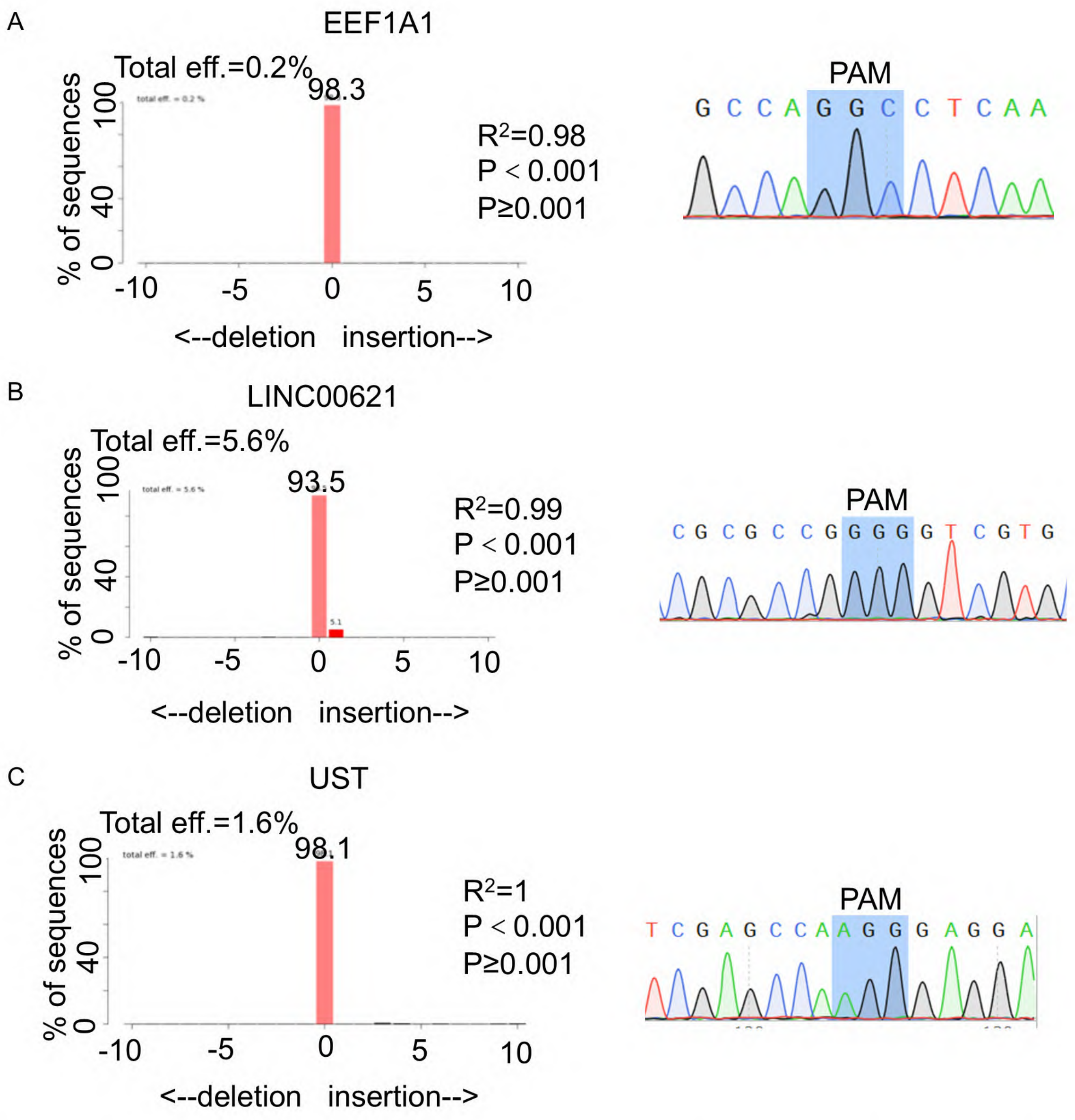

Figure S8 Detection of the off-target sites induced by SpCas9 with P53 sgRNA.

Figure S8 Detection of the off-target sites induced by SpCas9 with P53 sgRNA. (A-C) TIDE analysis and sequencing of off-target sides predicted by Tag-seq.

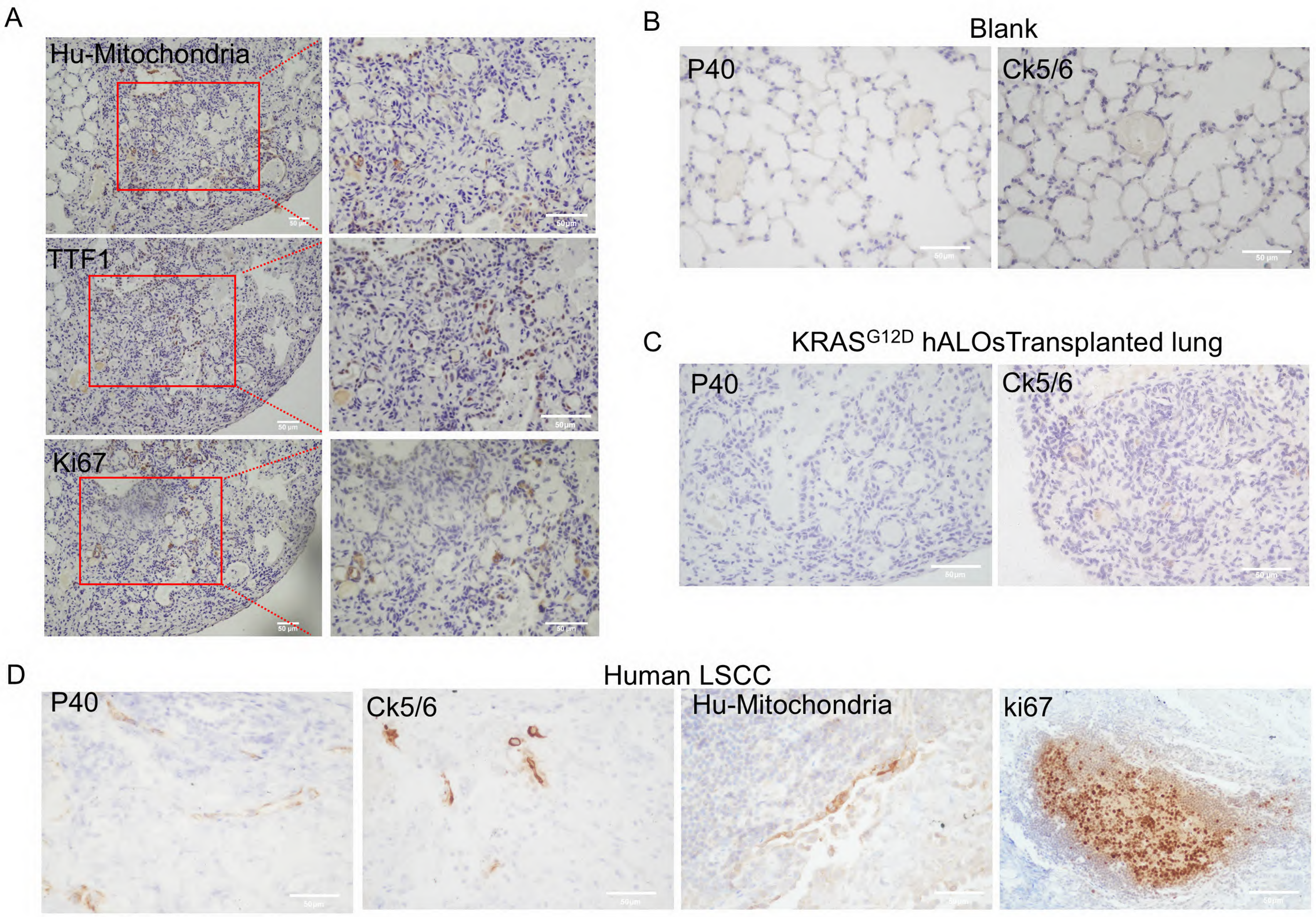

Figure S9 *In vivo* transplantation of KRAS^{G12D}-mutated hALOs.

Figure S9. *In vivo* transplantation of KRAS^{G12D}-mutated hALOs. (A) Immunohistochemistry staining of Human Mitochondria marker MAB1273, LUAD marker TTF1 and proliferation marker Ki67 in the same region of KRAS^{G12D} hALOs Transplanted lung. Scale bar, 50 μm. (B-C) Immunohistochemistry staining of LCC marker P40 and CK5&6 in blank (B) and KRAS^{G12D} hALOs Transplanted lung (C). Scale bar, 50 μm. (D) Immunohistochemistry staining of P40, CK5&6, Hu-Mitochondria and Ki67 in human LSCC sample. Scale bar, 50 μm.

Table S1. Sequence of used PCR primers for genotyping

Primer name	Primer sequence (5' to 3')	Note
eGFP-KI-F (5')	AACGGTGAAGGTGACAGCAG	eGFP knockin genotyping
eGFP-KI-R (5')	CCTCACATTGCCAAAAGACG	
eGFP-KI-F (3')	AGAACGTTCACGGCGACTAC	
eGFP-KI-R (3')	TGTGTGGACTTGGGAGAGGA	
mNeonGreen-KI-F (5')	AACGGTGAAGGTGACAGCAG	mNeonGreen knockin genotyping
mNeonGreen-KI-R (5')	CCTCACATTGCCAAAAGACG	
mNeonGreen-KI-F (3')	CGACTAGAGCTTGCGGAACC	
mNeonGreen-KI-R (3')	TGTGTGGACTTGGGAGAGGA	
TP53-F	AGGAGGTGCTTACGCATGTT	TP53 knockout genotyping
TP53-R	CCAGTTGCAAACCAGACCTC	
UST-F	TAAGGAAGGGGTTTGACGGG	UST genotyping
UST-R	CACTCGCTCTCATCGTGCAG	
LINC00621-F	TACACCACACCACGAGGCTC	LINC00621 genotyping
LINC00621-R	TGCTCTGTCTGCACCGCAAG	
EEF1A1-F	TATGGCCCTTGCGTGCCTTG	EEF1A1 genotyping
EEF1A1-R	ACCAGTGTGCAGATCTTGGC	
KRAS-WT-F	CCAGGCCTGCTGAAAATGAC	KRAS-G12D overexpression genotyping
KRAS-WT-R	ACTGGTCCCTCATTGCACTG	
KRAS-G12D-F	TACGATGTTCCAGATTACGC	
KRAS-G12D-R	ACTGGTCCCTCATTGCACTG	

Table S2. Sequence of used qPCR primers

Gene	Forward primer (5' to 3')	Reserve primer (5' to 3')
GAPDH	GGAGCGAGATCCCTCCAAAAT	GGCTGTTGTCATACTTCTCATGG
POU5F1	GGGAGATTGATAACTGGTGTGTT	GTGTATATCCCAGGGTGATCCTC
NKX2.1	CTCATGTTCATGCCGCTC	GACACCATGAGGAACAGCG
SFTPB	CTTCCAGAACCAGACTGACTCA	GCTCGGAGAGATCCTGTGTG
SFTPC	AGCAAAGAGGTCCTGATGGA	CGATAAGAAGGCGTTTCAGG
LAMP3	ACCGATGTCCAACTTCAAGC	TGACACCTTAGGCGGATTTT
AGER	GCCACTGGTGCTGAAGTGTA	TGGTCTCCTTTCCATTCCTG
PDPN	AACCAGCGAAGACCGCTATAA	CGAATGCCTGTTACACTGTTGA
HOPX	CCGTCTTCTCTCAGCCACAT	CCAAGCAAGCTTCTTCACCA
SOX2	TACAGCATGTCCTACTCGCAG	GAGGAAGAGGTAACCACAGGG
SOX9	AGCGAACGCACATCAAGAC	CTGTAGGCGATCTGTTGGGG
FOXQ1	CCTACTCGTACATCGCGCTCAT	TCGTTGAGCGAAAGGTTGTGGC
ETV4	AGGAACAGACGGACTTCGCCTA	CTGGGAATGGTCGCAGAGGTTT
MMP1	ATGAAGCAGCCCAGATGTGGAG	TGGTCCACATCTGCTCTTGGCA
MMP10	TCCAGGCTGTATGAAGGAGAGG	GGTAGGCATGAGCCAAACTGTG
FUT9	TGGAATCAGCCAGCTCTGTGCT	CGTTGTGAGATGGCATCCTTGG
CLIC2	CCAATCCTCCGTTCCTGGTGTA	ACTTGGGACTCAGGTGAGGGTA
AQP5	CTGTCCATTGGCCTGTCTGTC	GGCTCATACGTGCCTTTGATG
KRAS (total)	CAGTGCAATGAGGGACCAGT	CCTACTAGGACCATAGGTAC
KRAS (transgene)	TACGATGTTCCAGATTACGC	ACTGGTCCCTCATTGCACTG
ANKRD1	GCCCAGATCGAATTCCGTGA	ACGGGGTATCTCCTTCTCTGT
CYR61	TCGGCTGGTCAAAGTTACCG	CTCCATTCCAAAAACAGGGAGC
CTGF	GTGTGCACCGCCAAAGAT	AAACGTGTCTTCCAGTCGGT
VEGFA	CTCCACCATGCCAAGTGGT	GCAGTAGCTGCGCTGATAGA
PDGFA	CAGCGACTCCTGGAGATAGACT	CGATGCTTCTCTTCCTCCGAATG

Table S3. Antibodies Used in the Present Study

Primary Antibodies	Dilution rate	Manufacturer	Cat. No.
NKX2.1/TTF1	1:200	Abcam	ab76013
Anti-Mitochondria	1:30	EMD-Millipore	MAB1273
AQP5	1:150	Abcam	ab92320
PDPN	1:200	Abcam	ab10288
ki67	1:250	Abcam	ab16667
AGER	1:400	R&D systems	AF1145
Pro SP-C	1:200	Merck Millipore	AB3786
Mature-SFTPC	1:300	SEVEN HILLS	WRAB-76694
Cleaved Caspase-3	1:400	Cell Signaling Technology	#9664
LAMP3	1:200	Diaclone	857.770.000
P53	1:50	HuaBio	ET1601-13
P21	1:200	HuaBio	HA500156
ZO-1	1：100	Thermo Fisher Scientific	33-9100
E-cadherin	1:100	R&D systems	AF748
Napsin A	1：1	ZSGB-BIO	ZM-0473
CK5&6	1：1	ZSGB-BIO	ZM-0313
P40	1：1	ZSGB-BIO	ZA-0483
Dnokey anti-goat (RRX)	1:500	Jackson ImmunoResearch	705-295-147
Donkey anti-rabbit (Alexa488)	1:500	Thermo Fisher Scientific	A-21206
Donkey anti-mouse (Alexa647)	1:300	Thermo Fisher Scientific	A-31571
